# 1,520 reference genomes from cultivated human gut bacteria enable functional microbiome analyses

**DOI:** 10.1038/s41587-018-0008-8

**Published:** 2019-02-04

**Authors:** Yuanqiang Zou, Wenbin Xue, Guangwen Luo, Ziqing Deng, Panpan Qin, Ruijin Guo, Haipeng Sun, Yan Xia, Suisha Liang, Ying Dai, Daiwei Wan, Rongrong Jiang, Lili Su, Qiang Feng, Zhuye Jie, Tongkun Guo, Zhongkui Xia, Chuan Liu, Jinghong Yu, Yuxiang Lin, Shanmei Tang, Guicheng Huo, Xun Xu, Yong Hou, Xin Liu, Jian Wang, Huanming Yang, Karsten Kristiansen, Junhua Li, Huijue Jia, Liang Xiao

**Affiliations:** 1 BGI-Shenzhen, Shenzhen, China; 2 China National Genebank, BGI-Shenzhen, Shenzhen, China; 3 Laboratory of Genomics and Molecular Biomedicine, Department of Biology, University of Copenhagen, Copenhagen, Denmark; 4 Key Laboratory of Dairy Science, College of Food Sciences, Northeast Agricultural University, Harbin, Heilongjiang China; 5 BGI Education Center, University of Chinese Academy of Sciences, Shenzhen, China; 6 Shenzhen Engineering Laboratory of Detection and Intervention of Human Intestinal Microbiome, Shenzhen, China; 7 BGI-Qingdao, BGI-Shenzhen, Qingdao, China; 8 James D. Watson Institute of Genome Sciences, Hangzhou, China; 9 Qingdao-Europe Advanced Institute for Life Sciences, Qingdao, China; 10 School of Bioscience and Biotechnology, South China University of Technology, Guangzhou, China; 11 Macau University of Science and Technology, Taipa, Macau, China; 12 Department of Digestive Diseases, Huashan Hospital of Fudan University, Shanghai, China

**Keywords:** Microbiome, Bacterial genomics, Bacterial genetics, Metagenomics

## Abstract

Reference genomes are essential for metagenomic analyses and functional characterization of the human gut microbiota. We present the Culturable Genome Reference (CGR), a collection of 1,520 nonredundant, high-quality draft genomes generated from >6,000 bacteria cultivated from fecal samples of healthy humans. Of the 1,520 genomes, which were chosen to cover all major bacterial phyla and genera in the human gut, 264 are not represented in existing reference genome catalogs. We show that this increase in the number of reference bacterial genomes improves the rate of mapping metagenomic sequencing reads from 50% to >70%, enabling higher-resolution descriptions of the human gut microbiome. We use the CGR genomes to annotate functions of 338 bacterial species, showing the utility of this resource for functional studies. We also carry out a pan-genome analysis of 38 important human gut species, which reveals the diversity and specificity of functional enrichment between their core and dispensable genomes.

## Abstract (web summary)

A resource of >1,500 bacterial reference genomes sheds light on the human gut microbiome.

## Main

The human gut microbiota refers to the all the microorganisms that inhabit the human gastrointestinal tract. Diverse roles of the gut microbiota in human health and disease have been recognized^1,2^. Metagenomic studies have transformed our understanding of the taxonomic and functional diversity of human microbiota, but more than half of the sequencing reads from a typical human fecal metagenome cannot be mapped to existing bacterial reference genomes^3,4^. The lack of high-quality reference genomes has become an obstacle for high-resolution analyses of the human gut microbiome.

Although the previously reported Integrated Gene Catalog (IGC) has enabled metagenomic, metatranscriptomic and metaproteomic analyses^3,5,6^, the gap between compositional and functional analyses can only be filled by individual bacterial genomes. Genes that co-vary among samples can be clustered into metagenomic linkage groups^7^, metagenomic clusters^8^ and metagenomic species^9,10^, whose annotation depends on alignment to the limited number of existing reference genomes. Other metagenomics-based analyses of the gut microbiome—for example, single nucleotide polymorphisms (SNPs), indels and copy number variations—rely on the coverage and quality of reference genomes^11–13^.

Despite the rapid increase in the number of sequenced bacterial and archaeal genomes, reference genomes for gut bacteria are underrepresented. It is estimated that <4% of the bacterial genomes in the US National Center for Biotechnology Information (NCBI) database belong to the human gut microbiota. Rather, the focus has been on clinically relevant pathogenic bacteria, which are overrepresented in the microbial databases. The first catalog of 178 reference bacterial genomes for the human microbiota was reported by the Human Microbiome Project (HMP)^14^ in 2010. To date, the HMP has sequenced >2,000 microbial genomes cultivated from human body sites, 437 of which are gut microbiota (data accessed 8 September 2017). However, the number of reference gut bacterial genomes is still far from saturated.

We present a reference catalog of genomes of cultivated human gut bacteria (named the CGR), established by culture-based isolation of >6,000 bacterial isolates from fecal samples of healthy individuals. The CGR comprises 1,520 nonredundant, high-quality draft bacterial genomes, contributing at least 264 new reference genomes to the gut microbiome. After inclusion of CGR genomes, the mapping rate of selected metagenomic datasets improved from around 50% to over 70%. In addition to improving metagenomic analyses, the CGR will improve functional characterization and pan-genomic analyses of the gut microbiota at high resolution.

## Results

### Expanded catalog of gut bacterial genomes

We obtained 6,487 bacterial isolates from fresh fecal samples donated by 155 healthy volunteers by using 11 different media under anaerobic conditions (Supplementary Fig. 1a and Supplementary Table 1). Notably, more than half of the isolates were cultured from MPYG medium (Supplementary Fig. 1b). All the isolates were subjected to 16 S rRNA gene amplicon sequencing analysis, and 1,759 nonredundant isolates that provided broad coverage of the phylogenetic tree were selected for whole-genome sequencing (Supplementary Fig. 1c and Supplementary Table 2). After de novo assembly of the next-generation sequencing reads, we identified 104 isolates that contained more than one genome. These assembled sequences were then parsed into 212 genomes using our in-house pipeline (Supplementary Table 3). Briefly, multi-genomes were split at scaffold level on the basis of G + C content versus sequencing depth. The closest reference genomes for the spilt scaffolds were determined on the basis of average nucleotide identity (ANI), and the mis-split scaffolds were mapped back to their closest reference genome to get the final split genome (see Methods). In total, we obtained a collection of 1,867 newly assembled genomes, 1,520 (81.4%) of which fulfilled the HMP’s criteria for high-quality draft genomes and exceeded 95% genome completeness and less than 5% contamination as evaluated by CheckM. The genome sizes and G + C contents of CGR ranged from 0.2 to 7.9 Mbp and 26.56% to 64.28%, respectively. A total of 5,749,641 genes were predicted from the annotation data (Supplementary Table 4).

**Fig. 1:**
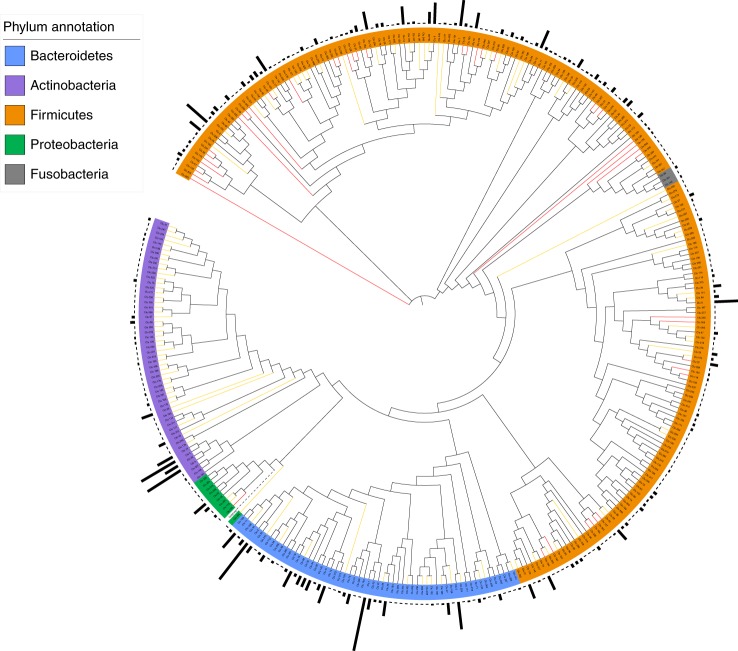
Phylogenetic tree of 1,520 isolated gut bacteria based on whole-genome sequences. The 1,520 high-quality genomes in CGR are classified into 338 species-level clusters (ANI ≥ 95%) based on their whole-genome sequences. Bacterial species from Firmicutes are colored in orange; Bacteroidetes, blue; Proteobacteria, green; Actinobacteria, violet; Fusobacteria, gray. Novel genera and species are highlighted by red and orange branches, respectively. The bar on the outermost layer indicates the number of genomes archived in each cluster. *Rhizobium selenitireducens* ATCC BAA 1503 was used as an outgroup for phylogenetic analysis.

Taxonomic annotation of CGR was carried out using a self-constructed, efficient ANI-based pipeline (Supplementary Fig. 2). The 1,520 high-quality genomes were classified into 338 species-level clusters (ANI ≥ 95%, a species delineation corresponding to 70% DNA–DNA hybridization), which covered all the major phyla of the human gut microbiota, including Firmicutes (211 clusters, 796 genomes), Bacteroidetes (60 clusters, 447 genomes), Actinobacteria (54 clusters, 235 genomes), Proteobacteria (10 clusters, 36 genomes) and Fusobacteria (3 clusters, 6 genomes) (Fig. 1a and Supplementary Table 5). Among these 338 clusters, 134 clusters (corresponding to 264 genomes) were not annotated to any present reference genomes in NCBI (Fig. 1a), and 50 clusters did not fall within any sequenced genera (Supplementary Table 5). To corroborate the presence of novel species in CGR, we carried out additional taxonomic identification using 16 S rRNA gene analysis. A species was recognized as novel if its 16 S rRNA gene sequence had < 98.7% similarity with known species in the EzBioCloud database (see Methods). Overall, we identified 350 distinct bacterial species (based on operational taxonomic units), including 149 candidate novel species, 42 of which represent candidate novel genera. These results underscore the value of the individual reference genomes provided by the CGR.

Despite the variation of individual microbiota at the genus level, the CGR identified bacterial populations with broad diversity, covering eight out of nine core genera in the Chinese gut microbiota^15^. More than 80 species were novel in comparison with the previously sequenced species from a reported 1,000 cultured bacterial species from the human gastrointestinal tract^16^ (Supplementary Fig. 3a). Moreover, the CGR successfully identified 38 genera that were of low relative abundance ( < 1%) according to the IGC^6^, which is a large catalog of reference genes derived from a collection of ~1,250 metagenomic samples from individuals on three continents (Supplementary Fig. 3b). Among them, 7 genera were identified with more than 20 genomes (*Bifidobacterium*, *Collinsella*, *Coprobacillus*, *Dorea*, *Streptococcus*, *Prevotella* and *Parabacteroides*). The CGR also identified another 9 genera that were not detected by IGC^6^ (*Butyricicoccus*, *Butyricimonas*, *Catenibacterium*, *Dielma*, *Erysipelatoclostridium*, *Megamonas*, *Melissococcus*, *Peptoclostridium* and *Vagococcus*) (Supplementary Fig. 3b). These results underscore the contribution of the CGR to the existing database of gut bacterial whole genomes.

### Improvement in metagenomic and SNP analyses

**Fig. 2:**
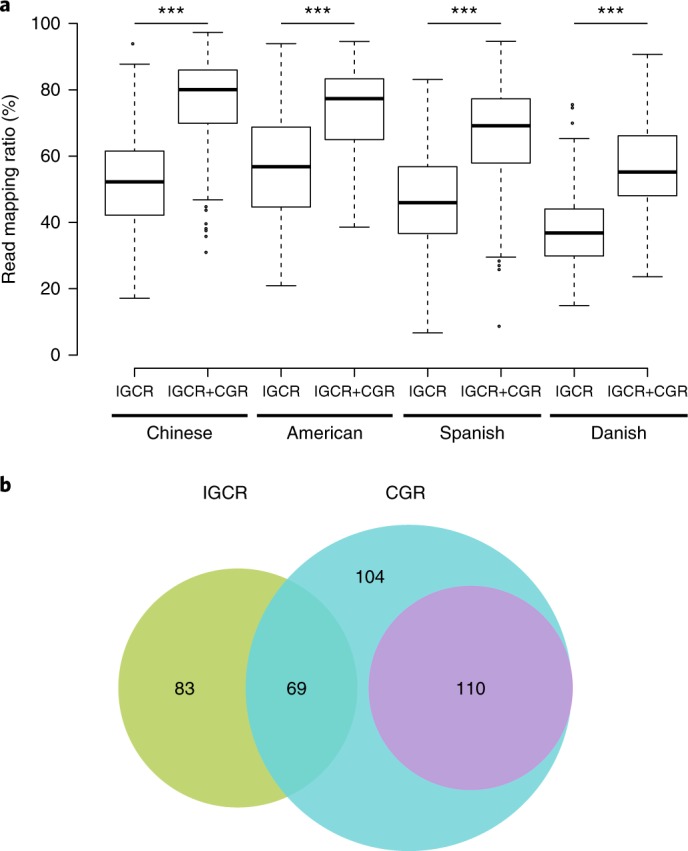
Contribution of CGR to metagenomic and SNP analyses. **a**, The read mapping ratio of a previous metagenomic analysis (IGCR) was significantly improved by CGR (IGCR + CGR) in fecal samples from Chinese (*n* = 368, *P* = 6 × 10^−78^), American (*n* = 139, *P* = 2 × 10^−17^), Spanish (*n* = 320, *P* = 4 × 10^−50^) and Danish (*n* = 109, *P* = 4 × 10^−17^) individuals. The significance of improvement was determined by two-side Wilcoxon rank-sum test. IGCR, 3,449 reference genomes used in the IGC study^6^; CGR, 1,520 reference genomes generated in this study. Each box plot illustrates the estimated median (center line), upper and lower quartiles (box limits), 1.5 × interquartile range (whiskers), and outliers (points) of the read mapping ratio. **b**, Reference genomes for SNP analysis generated in previous study^17^ (IGCR, green) and current study (CGR, blue). The unclassified species of reference genomes in CGR are highlighted in violet.

The existing reference genomes for metagenomic sequence mapping are far from saturated. For example, the genomes or draft genomes of bacteria and archaea used in a recent study allowed mapping of less than half of the sequences in the fecal metagenome^3,4^. To illustrate the value of the CGR to metagenomic analyses, we performed sequence mapping using previous metagenomic data^6^ with or without CGR. For Chinese samples, the read mapping rate in the original study that used the IGCR dataset (3,449 reference genomes from IGC^6^) was 52.00%, which was significantly improved to 76.88% after the inclusion of the CGR dataset (Fig. 2a and Supplementary Table 6). Since all the samples in the CGR were from China, it is reasonable to assume that this genome dataset contributes substantially to the Chinese fecal metagenome. To evaluate the contribution of the CGR to the mapping of non-Chinese metagenomes, we carried out a similar analysis using metagenomic data from American, Spanish and Danish fecal samples. Notably, the metagenomic read mapping ratios of these samples all increased substantially (Fig. 2a), although to a lesser extent compared with that of Chinese samples (Supplementary Fig. 4a). The improvement of mapping rates in both Chinese and non-Chinese samples indicates that the CGR covers a considerable number of gut bacterial species shared by people between these countries. To reveal the improvement of gene and protein diversity enabled by the CGR, we compared the gene and protein cumulative curve based on genomes used in a previous IGC study and after addition of the CGR (Supplementary Fig. 4b,c). The number of gene and protein families increased with inclusion of the first 1,500 genomes, but more or less plateaued at around 3,000 genomes. The addition of our CGR genomes led to a substantial increase in the number of added gene and protein families as a function of genome number. A total of 373,555 gene clusters and 149,945 protein clusters were added by inclusion of the CGR, corresponding to a 22% and 16% increase in known gene and protein sequence diversity, respectively.

To further illustrate the utility of the CGR, we used it to analyze gut microbiome SNPs in a cohort of 250 samples from the TwinsUK registry^17^. We generated a new set of 282 nonredundant representative genomes from the CGR (see Methods, Supplementary Fig. 5 and Supplementary Table 7), which number nearly doubled the 152 reference genomes used in the original TwinsUK analysis^17^. To highlight the new reference genomes identified by analysis with the existing genomes and the CGR genomes, we performed an ANI-based alignment of the 282 genomes with the previously reported 152 genomes. Among the 192 newly added reference genomes, 85 were classified species while 107 were unclassified species (Fig. 2b). A high SNP density was found in *Ruminococcus* sp. CAG:108 (Clu 21), unclassified Firmicutes (Clu 157), *Eubacterium rectale* (Clu 6), *Escherichia coli* (Clu 22), and *Ruminococcus* sp. CAG:57 (Clu 19), suggesting a high degree of variations in the genomes of these species, while *Lactobacillus gasseri* (Clu 241), *Enterococcus fecalis* (Clu 316), *Enterococcus durans* (Clu 274) and *Streptococcus mutans* (Clu 217) showed lower SNP density. A total of 9.14 million SNPs were identified. The number of SNPs was increased for some species due to the newly added high-quality reference genomes in the CGR. We conclude that the CGR is a valuable resource for metagenomic studies because of the significant improvement in metagenomic resolution it enables.

### Functions of gut microbiome bacteria

**Fig. 3:**
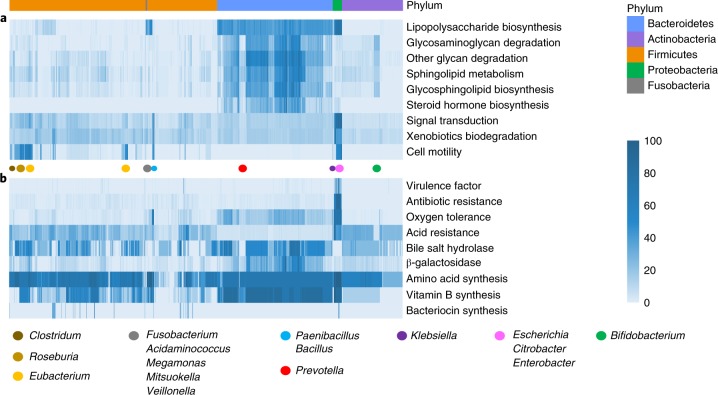
Functional landscape of gut microbiota. The gene abundance of listed functions in 1,520 genomes of CGR is indicated by the color depth in the heat map. The listed functions are enriched in specific phyla or genera (**a**) or might have deleterious or beneficial effects on human health (**b**). The bacterial species are ordered according to the phylogenetic tree in Fig. 1. The relative positions of phyla and genera in the phylogenetic tree are indicated by the colored ribbons and dots, respectively.

To better elucidate functions of the gut microbiota, we annotated gene functions in 1,520 CGR genomes using KEGG (the Kyoto Encyclopedia of Genes and Genomes)^18^. Functional pathways at KEGG level 2 showed that pathways involved in carbohydrate and amino acid metabolism are abundant in all isolated strains, suggesting that these are core functions of the gut microbiota (Supplementary Fig. 6). We also analyzed KEGG level 3 pathways and focused on those enriched at the phylum or genus level (Fig. 3a). We found that lipopolysaccharide biosynthesis (ko00540) genes were widely distributed in the phyla Fusobacteria, Bacteroidetes and Proteobacteria, the main phyla of gram-negative bacteria. Genes involved in glycan degradation (ko00531 and ko00511) were abundant in the genomes of the Bacteroidetes phylum. This observation is consistent with the notion that members of Bacteroidetes are prominent human gut symbionts that help degrade glycans in the diet and the gut mucosa^19^. The members of the Bacteroidetes also possess a high proportion of genes involved in sphingolipid metabolism (ko00600), glycosphingolipid biosynthesis (ko00601, ko00603 and ko00604) and steroid hormone biosynthesis (ko00140). Sphingolipids and hormone biosynthesis are ubiquitous in eukaryotic cells but not present in most bacteria. These results suggest that members of the Bacteroidetes not only participate in energy metabolism in the gut, but may also act in sphingolipid and hormone signaling in mammalian cells. The Proteobacteria showed relatively high abundance in genes involved in degradation of xenobiotics (ko01220), possibly contributing to the degradation of environmental chemicals and pharmaceuticals in the gut.

The signal transduction system (two-component system, ko02020) and xenobiotics degradation (KEGG level 2 pathway) were ubiquitous in the genera *Paenibacillus*, *Bacillus*, *Klebsiella*, *Escherichia*, *Citrobacter* and *Enterobacter*, which are also presented in environmental niches, such as soil and water. The abundant signal transduction and xenobiotics degradation systems allow these genera to sense and respond to various stresses and toxic substance presented in natural environments. Cell motility (chemotaxis, ko02030; flagellar assembly, ko02040) was conserved in the genera *Roseburia*, *Paenibacillus*, *Bacillus*, *Escherichia*, *Citrobacter* and *Enterobacter*, but varied within the genera *Clostridium* and *Eubacterium*.

Next we investigated functions and pathways that are annotated in the KEGG database, but not categorized as KEGG pathways (Fig. 3b and Supplementary Table 9). Virulence factors and antibiotic resistance genes were annotated using the Virulence Factors Database (VFDB)^20^ and Comprehensive Antibiotic Resistance Database (CARD)^21^, respectively. Virulence factors and antibiotic resistance are clinically relevant and are abundant in the Proteobacteria phylum, suggesting that this phylum may be a reservoir for opportunistic pathogens. We examined the distribution of genes involved in responses to stresses frequently encountered by gut bacteria: oxygen tolerance and acid resistance. Oxygen tolerance was reflected by the number of genes encoding catalase and superoxide dismutase, two detoxification enzymes that scavenge reactive oxygen species produced during aerobic respiration. As expected, the facultative anaerobic bacteria in the genera *Paenibacillus*, *Bacillus*, *Klebsiella*, *Escherichia*, *Citrobacter* and *Enterobacter* were more oxygen tolerant. In addition to the previously reported *Bacteroides fragilis*^22^, other members of Bacteroidetes also showed moderate oxygen tolerance. Notably, bacteria in the Bacteroidetes phylum and the *Bifidobacterium* genus generally lacked acid resistance genes, suggesting that potential probiotics based on these organisms may suffer impaired survival in the acidic stomach environment after oral administration. Finally, we examined the distribution of six bacterial functions in the CGR that might have beneficial effects on human health. Amino acid and vitamin B synthesis genes were widely present in various gut bacteria, suggesting that gut microbiota might be an alternative source for nutrients that are sparse in vegetarian diets. Genes encoding bacterial bile salt hydrolases, which transform primary bile acids into secondary bile acids in the human intestine, were also ubiquitous in most gut bacteria. Genes encoding β-galactosidases, which might attenuate problems associated with lactose intolerance, were relatively abundant in the phylum Bacteroidetes. Genes involved in bacteriocin synthesis in gut bacteria were relatively rare and did not show phylum- or genus-specific distribution.

### Core and pan-genomes of underrepresented gut bacteria

We carried out a pan-genome analysis of 36 species or clusters that contain more than ten genomes, as well as two other species enriched in healthy controls compared with patients with type 2 diabetes in previous studies^7,23,24^, *Fecalibacterium prausnitzii* (cluster 63, seven genomes) and butyrate-producing bacterium SS3_4 (cluster 45, nine genomes). These clusters covered the phyla Firmicutes, Bacteroidetes, Actinobacteria and Proteobacteria (Supplementary Fig. 7a and Supplementary Table 8a). The pan-genome of a cluster can be defined as the sum of the core genes and dispensable genes (including unique genes and accessory genes) of all the members within that cluster^25^. Our pan-genome analysis showed that *Eubacterium rectale* (cluster 37) contained the lowest proportion of core genes (12%); the remaining genes fell into accessory and unique genomes (38% and 40%, respectively). In contrast, *Eubacterium* 3_1 (cluster 6) contained the largest proportion of core genes (53%) (Supplementary Fig. 7b). The pan-genome fitting curves showed that most clusters in Bacteroidetes displayed an ‘open’ pan-genome and had a relatively large pan-genome size, with *Bacteroides vulgatus* having the largest pan-genome size at 14,970 genes (Supplementary Figs. 8 and 9 and Supplementary Table 8b). In contrast, members in the phylum Actinobacteria tend to represent a relatively ‘closed’ pan-genome, which was only slightly expanded by the addition of CGR genomes. These results suggest that gut bacterial genomes are variable in the Bacteroidetes phylum, less variable in the Firmicutes and Proteobacteria, and fairly conserved in the Actinobacteria.

**Fig. 4:**
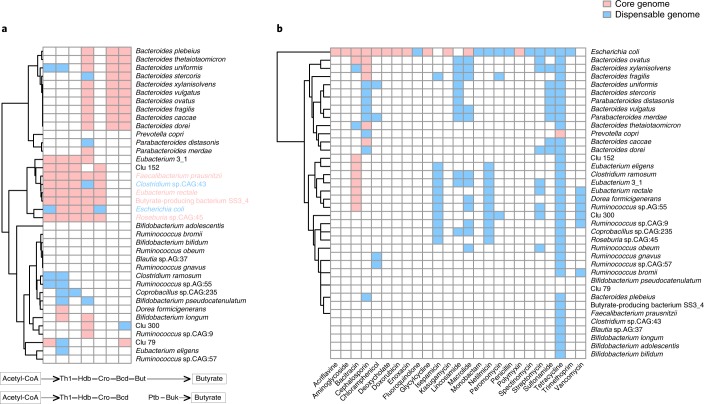
Pan-genome analysis of 38 representative clusters. **a**, The distribution of genes involved in butyrate biosynthesis pathway in the core genomes (pink) and dispensable genomes (cyan). The two pathways for butyrate biosynthesis from acetyl-CoA are shown below. The species with a complete butyrate biosynthesis pathway in the core genome and pan-genome are highlighted in pink and cyan, respectively. Thl, thiolase; Hdb, β-hydroxybutyryl-CoA dehydrogenase; Cro, crotonase; Bcd, butyryl-CoA dehydrogenase (including electron transfer protein α and β subunits); But, butyryl-CoA:acetate CoA transferase; Ptb, phosphate butyryltransferase; Buk, butyrate kinase. **b**, The distribution of ARGs in in the core genomes (pink) and dispensable genomes (cyan).

We also explored the distribution of genes involved in butyrate synthesis and antibiotic resistance in the pan-genomes of gut bacteria. Functional annotation showed that six clusters contained the complete acetyl-CoA to butyrate biosynthesis pathway (Fig. 4a). Among them, *F. prausnitzii*, *E. rectale*, butyrate-producing bacterium SS3_4 and *Roseburia* sp. CAG:45 harbored the complete pathway in their core genome, suggesting that the butyrate-producing function was highly conserved in these species. This result is consistent with the reported butyrate-producing capacity of these species^26–28^. To explore the distribution of antibiotic resistance within the established pan-genomes, we annotated 25 antibiotic resistance genes (ARGs) in each pan-genome. Consistent with a previous report^29^, the tetracycline resistance gene was widely present in the dispensable genome of these clusters (Fig. 4b). Notably, *Escherichia coli* contained almost all ARGs (23 of 25) in its pan-genome, with half of these present in the core genome (Fig. 4b). In contrast, *Bifidobacterium* species, including *B. bifibium*, *B. adolescentis*, *B. longum* and *B. pseudocatenulatum*, rarely contained ARGs in their pan-genomes.

To obtain a better understanding of the distribution of bacterial functions in the core and dispensable genomes, we annotated the genomes using the Clusters of Orthologous Groups (COG) database^30^. This revealed that several housekeeping functions were significantly enriched in the core genome, including post-translational modification, protein turnover and chaperones (O, *P* = 7.28 × 10^–12^); translation, ribosomal structure and biogenesis (J, *P* = 7.28 × 10^–12^); energy production and conversion (C, *P* = 7.28 × 10^–12^); amino acid transport and metabolism (E, *P* = 7.28 × 10^–12^); nucleotide transport and metabolism (F, *P* = 7.28 × 10^–12^); coenzyme transport and metabolism (H, *P* = 1.46 × 10^–11^); lipid transport and metabolism (I, *P* = 2.40 × 10^–10^); and inorganic ion transport and metabolism (P, *P* = 2.40 × 10^–10^) (Supplementary Fig. 10). By contrast, COG categories enriched in the dispensable genome included cell wall-membrane-envelope biogenesis (M, *P* = 2.70 × 10^–9^); cell motility (N, *P* = 3.11 × 10^–5^); signal transduction mechanisms (T, *P* = 0.00039); intracellular trafficking secretion and vesicular transport (U, *P* = 1.22 × 10^–7^); defense mechanisms (V, *P* = 7.28 × 10^–12^); transcription (K, *P* = 3.64 × 10^–11^); replication recombination and repair (L, *P* = 7.28 × 10^–12^); and function unknown (S, *P* = 0.03111). The remaining COG categories showed no significant differences in core and dispensable genome.

## Discussion

We used 11 culturing conditions for isolation of gut bacteria and archived more than 6,000 isolates. From this collection of isolates, we generated 1,520 high-quality draft reference genomes. The high coverage of the resulting CGR at the genus and species levels (including low-abundance species) demonstrates the value of culture-based methods for strain isolation from the gut microbiota. In line with this, a large number of gut bacterial species that were previously considered as unculturable have been successfully cultivated in two recent studies^31,32^. Although there was some overlap between the novel species archived by CGR and in these two studies, the CGR contains 659 additional genomes (representing 209 clusters or species). Our cultivation methods can be applied to expand the CGR until it is saturated with the genomes of culturable gut bacteria. After that, single-cell sequencing can be used to investigate genomes of unculturable bacteria, with an overall aim of defining a saturated set of reference genomes of gut microbiota to underpin a better understanding of gut microbiome biology.

We applied out CGR genome dataset to assign functions to gut bacteria. For example, we found that virulence factors and antibiotic resistance genes are enriched in *Klebsiella*, *Escherichia*, *Citrobacter* and *Enterobacter*, which are opportunistic pathogens frequently isolated in clinical samples^33^. The abundance of signal transduction and cell motility genes in these bacteria could further contribute to their pathogenicity^34,35^. Notably, the Proteobacteria also possess abundant genes for degradation of xenobiotics, which might affect drug metabolism of patients in drug therapy. In line with this, a recent study reported that intratumor Proteobacteria can metabolize chemotherapeutic drugs into inactive forms and thus attenuate the efficacy of cancer therapies^36^. The genes involved in beneficial functions such as glycan degradation and vitamin B synthesis are enriched in the *Bacteroides* genus, consistent with its mutualistic role in the human gut. Notably, we found that *Bacteroides* species contain a considerable number of genes involved in sphingolipid and steroid hormone synthesis, suggesting their potential for modulating signaling in mammalian cells. In support of this, a recent study reported that *Bacteroides fragilis* can take advantage of sphingolipid signaling to enable symbiosis in the intestine^37^. It is noteworthy that genes involved in glycan degradation and sphingolipid metabolism were also enriched in the genus *Bifidobacterium*, another well-known gut commensal microbe. However, genes involved in both pathways were not abundant in the *Prevotella* genus, suggesting a distinct function of *Prevotella* compared with other members of the Bacteroidetes phylum. This might account for observed negative correlations between the relative abundances of *Prevotella* and *Bacteroides* in the gut microbiota^38^. The potential role of gut bacteria in metabolism of estrogens has long been recognized^39^, but detailed mechanistic studies are still lacking. It will be interesting to explore the implication of this unique function of gut bacteria in hormone-related health or disease. The CGR also enabled the identification of several potential bacteriocin-producing bacteria strains, which merit further verification.

The CGR will improve metagenomic analyses, genome variation analyses, functional characterization and pan-genome analyses. The isolated gut bacteria strains have been deposited in the China National GeneBank (CNGB) and may be useful for studies that aim to alter microbiota functions, as novel probiotics, or for verification of disease-associated bacterial markers.

## Methods

### Anaerobic cultivation of fecal bacteria

Fecal samples were collected from 155 healthy human donors not taking any drugs during the last month before sampling. Detailed information is given in Supplementary Table 2. The samples were immediately transferred to an anaerobic chamber (Bactron Anaerobic Chamber, Bactron IV-2, Shellab, USA), homogenized in pre-reduced phosphate buffered saline (PBS) supplemented with 0.1% cysteine, and then diluted and spread on agar plates with different growth media (Supplementary Table 1). Plates were incubated under anaerobic condition in an atmosphere of 90% N_2_, 5% CO_2_ and 5% H_2_ at 37 °C for 2–3 d. Single colonies were picked and streaked onto new plates to obtain single clones. All the strains were stored in a glycerol suspension (20%, v/v) containing 0.1% cysteine at –80 °C. The collection of the 155 samples was approved by the Institutional Review Board on Bioethics and Biosafety of BGI under number BGI-IRB17005-T1. All protocols were in compliance with the Declaration of Helsinki and explicit informed consent was obtained from all participants. Bacteria in the CGR (Culturable Genome Reference) are deposited in and are available from the E-BioBank (EBB) of the China National GeneBank (http://ebiobank.cngb.org/index.php?g=Content&m=Hql&a=sample_5&id=393).

### Whole-genome sequencing and de novo assembly

#### DNA extraction

Isolates cultivated to stationary phase were centrifuged at 7,227*g* at 4 °C for 10 min, and the resulting pellets were resuspended in 1 ml of Tris-EDTA. For bacterial cell lysis, 50 µl of 10% SDS and 10 µl of proteinase K (20 mg/ml) were added, and the solution was incubated at 55 °C in a water bath for 2 h. The released genomic DNA was extracted using the phenol-chloroform method^40^.

#### Genome sequencing and assembly

Paired-end libraries with an insert size of 500 bp were constructed and sequenced on Illumina Hiseq 2000 platform to obtain about 100 × clean data for each sample. The reads were assembled using SOAPdenovo 2.04^41^ to form scaffolds from which the rRNA genes were extracted by RNAMMer 1.2^42^. An in-house pipeline was used to obtain the best assembly containing an orthogonal design to investigate L,M,d,D,L,u,G (arguments of SOAPdenovo) and a single-factor design to investigate K (argument of SOAPdenovo) by comprehensively considering contig average length, longest scaffold and rRNA score. Libraries with an insert size of 240 bp were constructed and sequenced on the ionProton platform, which produced about 100 × clean data for each sample. The reads were assembled through SPAdes (version 3.1.0)^43^ to form scaffolds.

#### Assessment of genome quality

Six high-quality draft assembly criteria from the Human Microbiome Project (HMP)^14^ and rRNA (5 s, 16 s and 23 s) completeness were adopted to ensure the assembly quality. The criteria are (i) 90% of the genome assembly must be included in contigs > 500 bp, (ii) 90% of the assembled bases must be at > 5 × read coverage, (iii) the contig N50 must be > 5 kb, (iv) scaffold N50 must be > 20 kb, (v) average contig length must be > 5 kb, and (vi) > 90% of the core genes^44,45^ must be present in the assembly.

### Splitting for multi-genome isolates

The multi-genomes in isolates were initially identified using CheckM^46^ (contamination > 5%) and confirmed by manual inspection of the plot of G + C percentage vs. sequencing depth. An in-house pipeline was developed to split the scaffolds of multi-genomes into single genomes. Briefly, scaffolds in multi-genomes were first split on the basis of G + C percentage vs. sequencing depth values using the dbscan function of R (package “fpc”). The “complete” and “contamination” of split genomes were checked using CheckM. For split genomes with “complete” > 100% or “contamination” > 15%, an additional species-designating pipeline was used to obtain their closest reference (with ANI value > 90%). Finally, the mis-split scaffolds in each split genome were mapping back to the closest reference genome using BLASTn (-e 1e-5 -F F -m 8, blastn hits’ length > 90 nt, query scaffold coverage ≥ 50%) to obtain the final split genomes.

### Massive species and genus assignment process

#### NCBI-retrieved prokaryotic genomes

All complete genomes (update time 19 November 2014) and draft genomes (update time 8 August 2014) on the NCBI ftp site were downloaded to a local server. Items with more than one NCBI taxonomy identifier (taxid) or genome sequence not available or of non-prokaryotic source were removed, and of redundant items, only one was kept. As a result, 24,552 genomes, 19,116 genome-scale amino acid sequences, and their taxonomic information were obtained.

#### Average nucleotide identity (ANI)^47^ for species level taxonomic assignment

The taxonomic assignment of each query genome was determined by the taxonomic information of all the NCBI-available prokaryotic genomes. The tetra-base signature profiles of all the genomes and each query genome were acquired. A Pearson correlation test was performed between each query genome and all the genomes, resulting in a reference list sorted by decreasing correlation coefficient for each query genome. Then pairwise ANI alignment was performed between query and reference genomes one by one according to the reference list (tetra-base profile’s Pearson correlation test: correlation coefficient > 0 and *P* < 0.001) until the ANI value was larger than 95% in the top 500 items (defined as assigned in this case) or reference item number exceeded 500 without any ANI value being larger than 95% (defined as not assigned in this case).

#### Percentage of conserved proteins (POCP)^48^ for genus-level taxonomic assignment

The taxonomic assignment of each query genome was determined by the taxonomic information of all the NCBI-available prokaryotic genomes. The tetra-base signature profiles of all the reference genomes and the query genomes with no species assignment based on ANI were acquired. A Pearson correlation test was performed between each query genome and all the reference genomes, resulting in a reference list sorted by decreasing correlation coefficient for each query genome. Then the POCP calculation was performed between query and reference genomes one by one according to the reference list until the POCP value was larger than 50% in the top 500 items (defined as “assigned” in this case) or reference items number exceeded 500 without any POCP value being larger than 50% (defined as “not assigned” in this case).

#### 16S rRNA sequence analysis and novel species determination

16 S rRNA gene sequences were extracted from the isolate genomes using RNAmmer^42^, except for 16 genomes where extraction failed. The sequences were quality-control processed in EzBioCloud (http://www.ezbiocloud.net)^49^. The species-level operational taxonomic units (OTUs) were classified using mothur^50^ with an identity of 98.7% as a species-level cut-off, and cut-offs of 94.5% and 86.5% were used for genera and families^51^, respectively.

#### Comparison of CGR with genome datasets from other studies

To compare the new genomes and novel species archived in CGR with those identified in two recent studies, we downloaded 215 genomes reported by Browne et al.^32^ and 169 genomes reported by Lagier et al.^31^. We adopted a similar ANI pipeline as described above for species-level comparison by replacing the NCBI references with these newly downloaded genomes. “Map” was defined if the pairwise ANI value between a query genome in our 1,520 high-quality genomes and any one of references genomes (tetra-base profile’s Pearson correlation test: correlation coefficient > 0 and *P* < 0.001) was larger than 95%; if not, the species was defined as “unmap.”

### Construction of species clusters

Pairwise ANI alignment was performed among the 1,520 high-quality genomes, and then hclust from the R package was used for hierarchical clustering with distance of 0.05 (equivalent to 95% ANI). A set of 40 universally conserved single-copy genes encoding proteins in bacteria and archaea was used for construction of a phylogenetic tree. Marker genes were detected and aligned using specI^52^ and prank^53^. Alignments were trimmed by trimal^54^ and concatenated with in-house scripts. A phylogenetic tree was inferred using the maximum likelihood method with RAxML (version 8.2.8)^55^ for the clusters’ representative genomes (N50 longest among cluster) with *Rhizobium selenitireducens* ATCC BAA 1503 (taxoid:1336235) as an outgroup, and was visualized in iTOL (http://itol.embl.de/)^56^ online.

### Genome function annotation

The 1,520 high-quality genomes were functionally annotated. Genes were identified using Genemark^57^. The translated amino acid sequences of coding genes were aligned with RAPSearch (-s f -e 1e-2 -v 100 -u 2)^58^ against the Kyoto Encyclopedia of Genes and Genomes (KEGG version 76)^18,59^ (query match length higher than 50%) or with BLASTp (-e 1e-2 -F T -b 100 -K 1 -a 1 -m 8) against the Antibiotic Resistance Genes Database (ARDB) (both query and subject match length higher than 40%, with identity higher than the ARDB-recommended thresholds)^60^, the Virulence Factor Database (VFDB)^20,61^ (query match length higher than 50%, with identity higher than 60%), and the bacteriocin database (downloaded from BAGEL3^62^, with identity higher than 60%). Annotation of genes against the Comprehensive Antibiotic Resistance Database (CARD)^21^ was performed using Resistance Gene Identifier available as a downloadable command-line tool in the download section of the CARD website using default parameters.

### Mapping ratio of metagenomic samples

The metagenomic reads^6^ were first aligned to the reference genomes of IGCR (3,449 sequenced prokaryotic genomes from IGC^6^) using SOAP2^63^ (default parameters, except -m 100 -x 1000 -r 1 -l 30 -v 5 -c 0.95 -u). The unmapped reads were then aligned to the newly sequenced genomes of CGR. The read mapping ratio of different samples was calculated, and the difference between samples was determined by Wilcoxon test in R.

### Analysis of gene and protein diversity

#### Gene clusters

5,749,641 genes in the 1,759 CGR genomes and 11,330,042 genes in 3,449 IGCR genomes were clustered using CD-HIT^64^ with default parameters, except -G 0 -aS 0.9 -c 0.95 -M 0 -d 0 -r 1 -g 1, which amounts to 95% local sequence identity over 90% alignment coverage for the shorter sequence. A cluster is composed of two or more genes. An accumulative curve of gene clusters was drawn according to the sample name alphabetically with IGCR at the front part and CGR at the latter part.

#### Protein clusters

5,749,641 protein sequences translated from genes in the 1,759 CGR genomes and 11,330,042 protein sequences translated from genes in 3,449 IGCR genomes were clustered using the kClust algorithm^65^ with default parameters, which amounts to 20–30% maximum pairwise sequence identity over 80% alignment length with the longest sequence or seed of the cluster. A cluster is composed of two or more protein sequences. An accumulative curve of protein clusters was drawn according to the sample name alphabetically with IGCR at the front part and CGR at the latter part.

### SNP identification and similarity score

1,520 genomes from the CGR were aligned with the sequenced reads from the 250 TwinsUK samples using SOAP2 with identity ≥90%. Representative genomes used for SNP analysis were identified according to three criteria described previously^17^. The resulting 282 genomes (Supplementary Table 7) that fulfilled these criteria were used as references for SNP calling using SAMtools (frequency > 1% and supported by ≥4 reads) as previously described^10,17,20^. The reference genomes used in a previous study^18^ (152 genomes) were compared with that from CGR of this study (282 genomes) to identify shared and new reference genomes using ANI ≥ 95% as a threshold (species level).

### Pan genome analysis for 38 cluster

Clusters containing more than ten genomes (from CGR and NCBI), as well as *Fecalibacterium prausnitzii* (seven genomes) and butyrate-producing bacterium SS3_4 (nine genomes), were used for pan-genome analysis using the Bacterial Pan Genome Analysis tool (BPGA) pipeline^66^. The set of genes shared by all the members of cluster was defined as core genes, while genes partially shared in members (accessory genes) and unique to single members (unique genes) in a cluster were defined as dispensable gene^67^. The pan-genome fitting curves of 38 clusters were generated by the BPGA workflow and plotted in R (v.3.3.3). The functions of genes in the pan-genomes of 38 clusters were annotated by KEGG and ARDB, using arguments identical to those used for functional annotation of genomes. The acetyl-CoA-to-butyrate biosynthesis pathway was generated according to a previous study^68^, and the associated enzymes were identified according to the functional annotation and BLAST to the NCBI protein database (cut-off 1e–5, identity ≥70%, coverage ≥70%). The COG database^30^ was also used to identify the functional distribution in the core and dispensable sections via the BPGA pipeline. The significance of the difference between COG distribution in core and dispensable genomes was examined using Wilcoxon test as implemented in R (v.3.3.3).

### Reporting Summary

Further information on research design is available in the Nature Research Reporting Summary linked to this article.

## Online content

Any methods, additional references, Nature Research reporting summaries, source data, statements of data availability, and associated accession codes are available at 10.1038/s41587-018-0008-8.

## Supplementary Information

### Integrated supplementary information

Supplementary Figure 1 Cultivation and genome sequencing of the gut microbiota. (a) The 155 feces samples from healthy volunteers grouped by ages and sex. (b) The number of isolates achieved by 11 different culture media under anaerobic condition. (c) The workflow of the cultivation and sequencing of isolated gut bacteria.

Supplementary Figure 2 Workflow for species annotation of sequenced genomes. Species assignment was carried out using an average-nucleotide identity (ANI)-based pipeline. Genomes not assigned by ANI were subjected to genus annotation by POCP.

Supplementary Figure 3 Diversity and novelty of gut bacterial genomes archived in CGR. (a) The number of bacterial species archived in CGR belonging to 9 core genera of the human gut microbiota in Chinese. The archived bacterial species were compared to the previously reported 1,000 cultured bacterial species in the human gastrointestinal tract, with the known species shown in white and novel species shown in black. (b) Low abundance (<1%) gut bacterial genera identified in CGR. Grey box indicates the relative abundance of each genus from 1267 samples, according to the previous IGC study. Red dot indicates the number of species in each genus archived in this study. Each boxplot illustrates the estimated median (centre line), upper and lower quartiles (box limits), 1.5 × interquartile range (whiskers).

Supplementary Figure 4 The improvement in metagenomic analysis by CGR. (a) The improvement of reads mapping ratio in metagenomic analysis by CGR (relevant to Fig. 2a). The percentage of improvement is calculated by the following formula: (CGR-ICG)/(100-ICG). The percentage of improvement for Chinese (n=368) is significantly higher than American (n=139, *P*=8×10^-20^), Spanish (n=320, *P*=9×10^-33^), and Danish (n=109, *P*=2×10^-31^) individuals. The significance of improvement was determined by unpaired Wilcoxon rank-sum test (two.sided). ICG represents the reads mapping ratio calculated from 3,449 reference genomes (ICGR in Fig. 2a), CGR represents the reads mapping ratio calculated from the addition of 1,520 reference genomes (ICGR+CGR in Fig. 2a). Each boxplot illustrates the estimated median (centre line), upper and lower quartiles (box limits), 1.5 × interquartile range (whiskers), and outlier (points) of the reads mapping ratio. (b)(c) Gene and protein sequence diversity increased by CGR. Increase in number of new gene families (b) and protein families (c) across added genomes from ICGR (blue) and CGR (red).

Supplementary Figure 5 SNP density in the 282 reference genomes with a cumulative coverage of at least 10× in the 250 samples from the TwinsUK registry. The reference genomes are ordered according to the cumulative coverage, with new reference genomes generated by this study highlighted in red.

Supplementary Figure 6 Functional annotation of 1,520 genomes in CGR. The gene functions in the genomes are annotated using KEGG pathways, with level 2 functions shown in the figure. The stack bar on the out-most layer represents the number of genes with given functions in each genome. The phylogenetic tree is plotted according to Fig. 1.

Supplementary Figure 7 Statistics for the pan-genome analysis of the 38 clusters. (**a**) Genomes for each cluster used in the pan-genome analysis. (**b**) Composition of core genes, unique genes, and accessory genes in the genomes of the 38 clusters. The clusters were ordered by the proportion of core genes.

Supplementary Figure 8 Pan-genome fitting curves of the 38 clusters. The pan-genome fitting curves of 38 representative clusters, from Firmicutes (orange), Bacteroidetes (blue), Actinobacteria (violet), Proteobacteria (green), and Fusobacteria (grey). The pan-genome size is accumulated from all combinations of strains contained in each cluster.

Supplementary Figure 9 Pan- and core-genome analysis of the 38 clusters. The number of gene families in the pan (cyan) and core (pink) genomes are plotted as a function of the number of genomes of the 38 clusters. Box plots indicate 25 th and 75 th percentiles with medians shown as horizontal lines and whiskers set at 10 th and 90 th percentiles.

Supplementary Figure 10 COG distribution in the core genome and the dispensable genome. The percentage of 20 COGs in the core genome (pink) was compared to that in the pan-genomes (cyan) of 38 clusters. The significance of improvement was determined by two-side Wilcoxon rank-sum test (*,*P*< 0.05; **, *P* < 0.01; ***, *P* < 0.001). The exact *P* value is 0.931 for D, 2.70×10^-9^ for M, 3.11×10^-5^ for N, 7.28×10^-12^ for O, 3.88×10^-4^ for T, 1.22×10^-7^ for U, 7.28×10^-12^ for V, 7.28×10^-12^ for J, 3.64×10^-11^ for K, 7.28×10^-12^ for L, 7.28×10^-12^ for C, 0.261 for G, 7.28×10^-12^ for E, 7.28×10^-12^ for F, 1.46×10^-11^ for H, 2.40×10^-10^ for I, 0.874 for Q, 2.40×10^-10^ for P, 0.365 for R, and 0.031 for S. Each boxplot illustrates the estimated median (centre line), upper and lower quartiles (box limits), 1.5 × interquartile range (whiskers), and outlier (points) of the COG percentage.

### Supplementary information

Supplementary Text and Figures Supplementary Figures 1–10

Reporting Summary

Supplementary Table 1 Culture media used for isolation of gut bacteria

Supplementary Table 2 Information for the 6,487 isolates and 1,759 sequenced strains

Supplementary Table 3 List of separated genomes

Supplementary Table 4 Statistics for sequencing data of 1,867 strains

Supplementary Table 5 Taxonomic information for 1,520 genomes

Supplementary Table 6 Improvement of metagenomic reads mapping ratio by CGR

Supplementary Table 7 Improvement of SNP analysis by CGR

Supplementary Table 8 Pan-genome analysis of the 38 clusters

Supplementary Table 9 The function annotation of 1,520 genomes

## Data Availability

The assembly draft genomes and annotation information for the 1,520 CGR strains are deposited in the NCBI under accession code PRJNA482748, and these data are also available in the China National GeneBank (CNGB) Nucleotide Sequence Archive (CNSA; accession code CNP0000126). All bacterial strains in the CGR have been deposited in the CNGB, a nonprofit, public-service-oriented organization in China. The accession code for each strain is given in Supplementary Table 5 (Genebank_id). Researchers can explore strain information and request strains via http://ebiobank.cngb.org/index.php?g=Content&m=Hql&a=sample_5&id=393#.
