# Supplementary figures and images for: 1,520 reference genomes from cultivated human gut bacteria enable functional microbiome analyses
Source: Nat Biotechnol. 2019 Feb 4;37(2):179–85. doi: 10.1038/s41587-018-0008-8 (PMC6784896; doi:10.1038/s41587-018-0008-8)

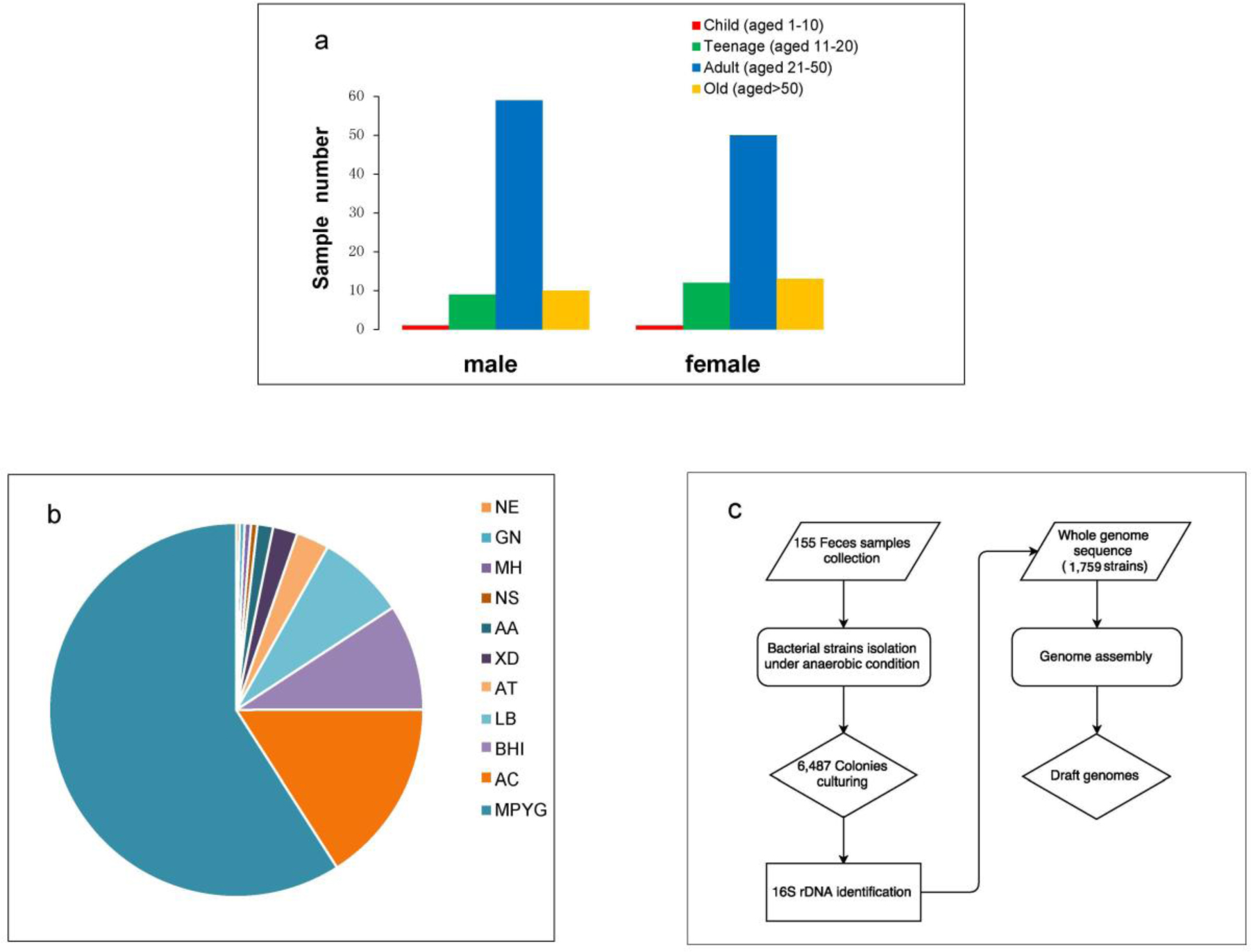

Supplement: Cultivation and genome sequencing of the gut microbiota. — (a) The 155 feces samples from healthy volunteers grouped by ages and sex. (b) The number of isolates achieved by 11 different culture media under anaerobic condition. (c) The workflow of the cultivation and sequencing of isolated gut bacteria. [file 41587_2018_8_Fig5_ESM.jpg]

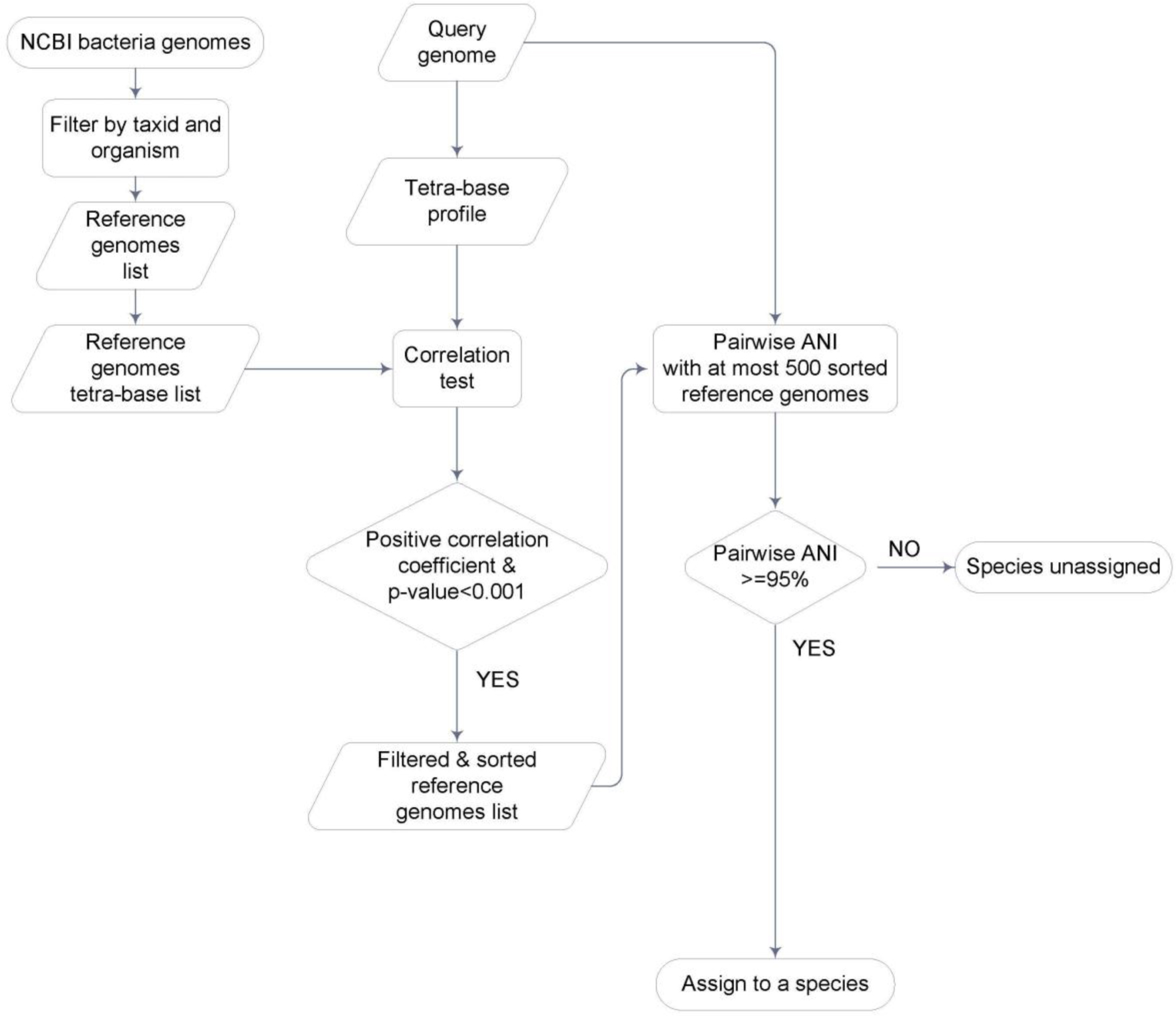

Supplement: Workflow for species annotation of sequenced genomes. — Species assignment was carried out using an average-nucleotide identity (ANI)-based pipeline. Genomes not assigned by ANI were subjected to genus annotation by POCP. [file 41587_2018_8_Fig6_ESM.jpg]

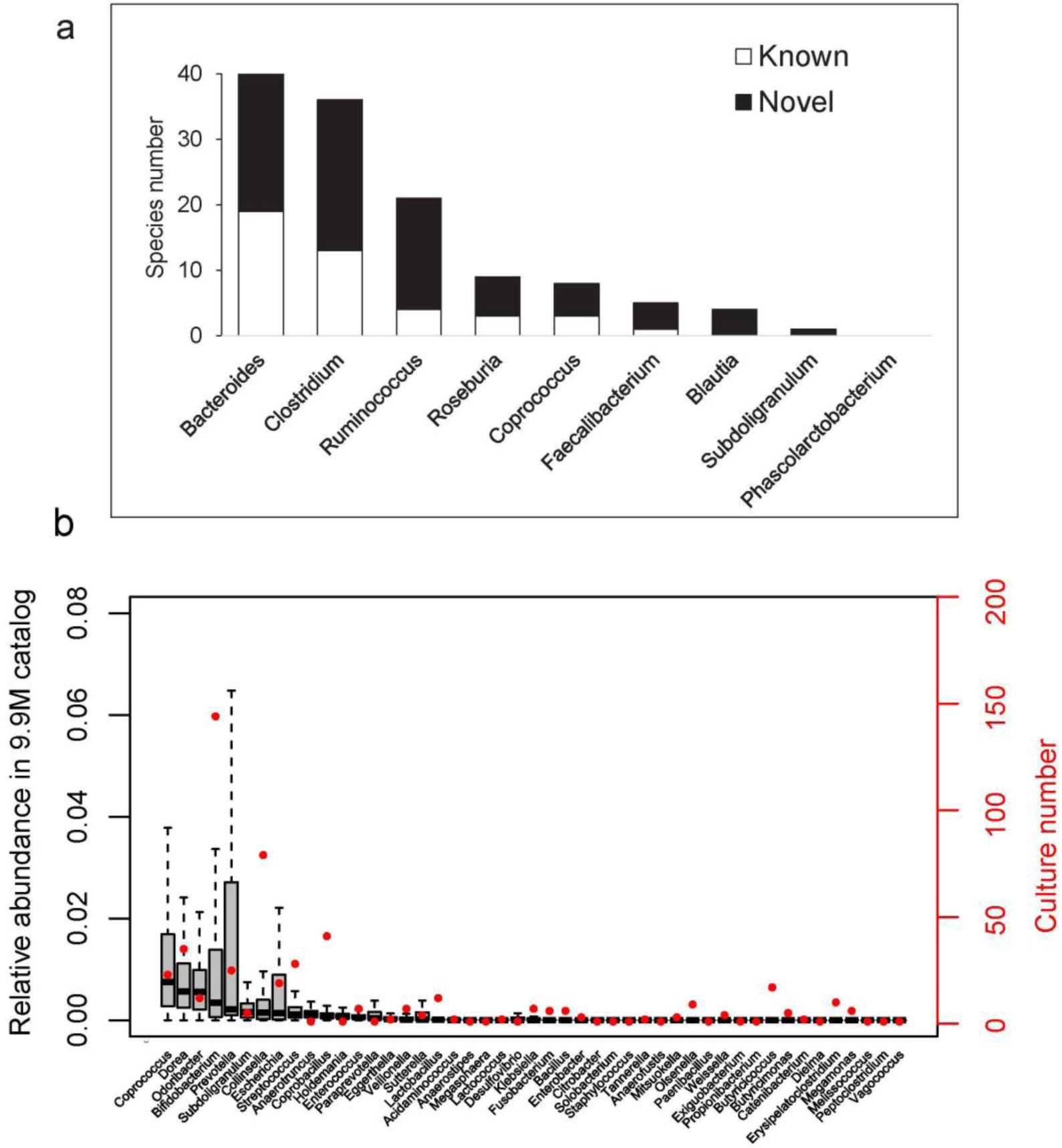

Supplement: Diversity and novelty of gut bacterial genomes archived in CGR. — (a) The number of bacterial species archived in CGR belonging to 9 core genera of the human gut microbiota in Chinese. The archived bacterial species were compared to the previously reported 1,000 cultured bacterial species in the human gastrointestinal tract, with the known species shown in white and novel species shown in black. (b) Low abundance (<1%) gut bacterial genera identified in CGR. Grey box indicates the relative abundance of each genus from 1267 samples, according to the previous IGC study. Red dot indicates the number of species in each genus archived in this study. Each boxplot illustrates the estimated median (centre line), upper and lower quartiles (box limits), 1.5 × interquartile range (whiskers). [file 41587_2018_8_Fig7_ESM.jpg]

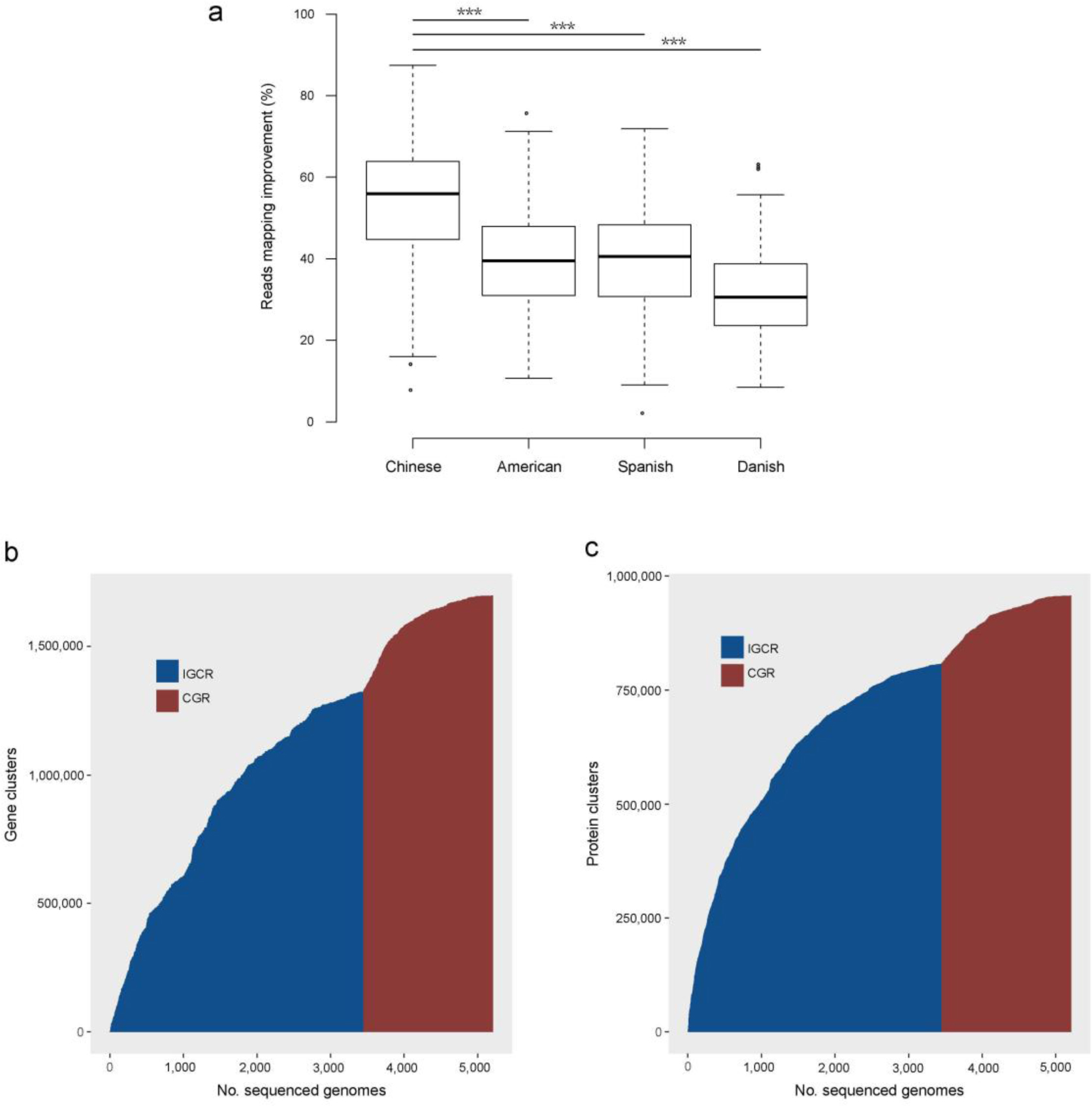

Supplement: The improvement in metagenomic analysis by CGR. — (a) The improvement of reads mapping ratio in metagenomic analysis by CGR (relevant to Fig. 2a). The percentage of improvement is calculated by the following formula: (CGR-ICG)/(100-ICG). The percentage of improvement for Chinese (n=368) is significantly higher than American (n=139, P=8×10^-20^), Spanish (n=320, P=9×10^-33^), and Danish (n=109, P=2×10^-31^) individuals. The significance of improvement was determined by unpaired Wilcoxon rank-sum test (two.sided). ICG represents the reads mapping ratio calculated from 3,449 reference genomes (ICGR in Fig. 2a), CGR represents the reads mapping ratio calculated from the addition of 1,520 reference genomes (ICGR+CGR in Fig. 2a). Each boxplot illustrates the estimated median (centre line), upper and lower quartiles (box limits), 1.5 × interquartile range (whiskers), and outlier (points) of the reads mapping ratio. (b)(c) Gene and protein sequence diversity increased by CGR. Increase in number of new gene families (b) and protein families (c) across added genomes from ICGR (blue) and CGR (red). [file 41587_2018_8_Fig8_ESM.jpg]

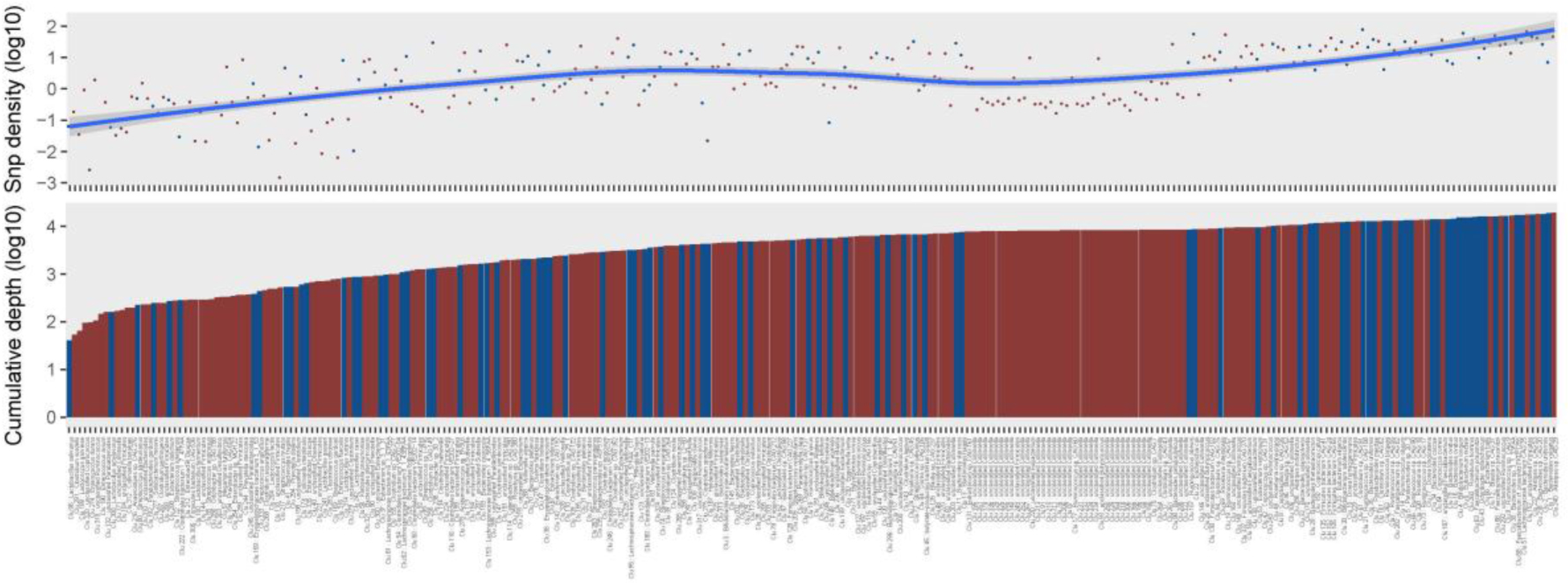

Supplement: SNP density in the 282 reference genomes with a cumulative coverage of at least 10× in the 250 samples from the TwinsUK registry. — The reference genomes are ordered according to the cumulative coverage, with new reference genomes generated by this study highlighted in red. [file 41587_2018_8_Fig9_ESM.jpg]

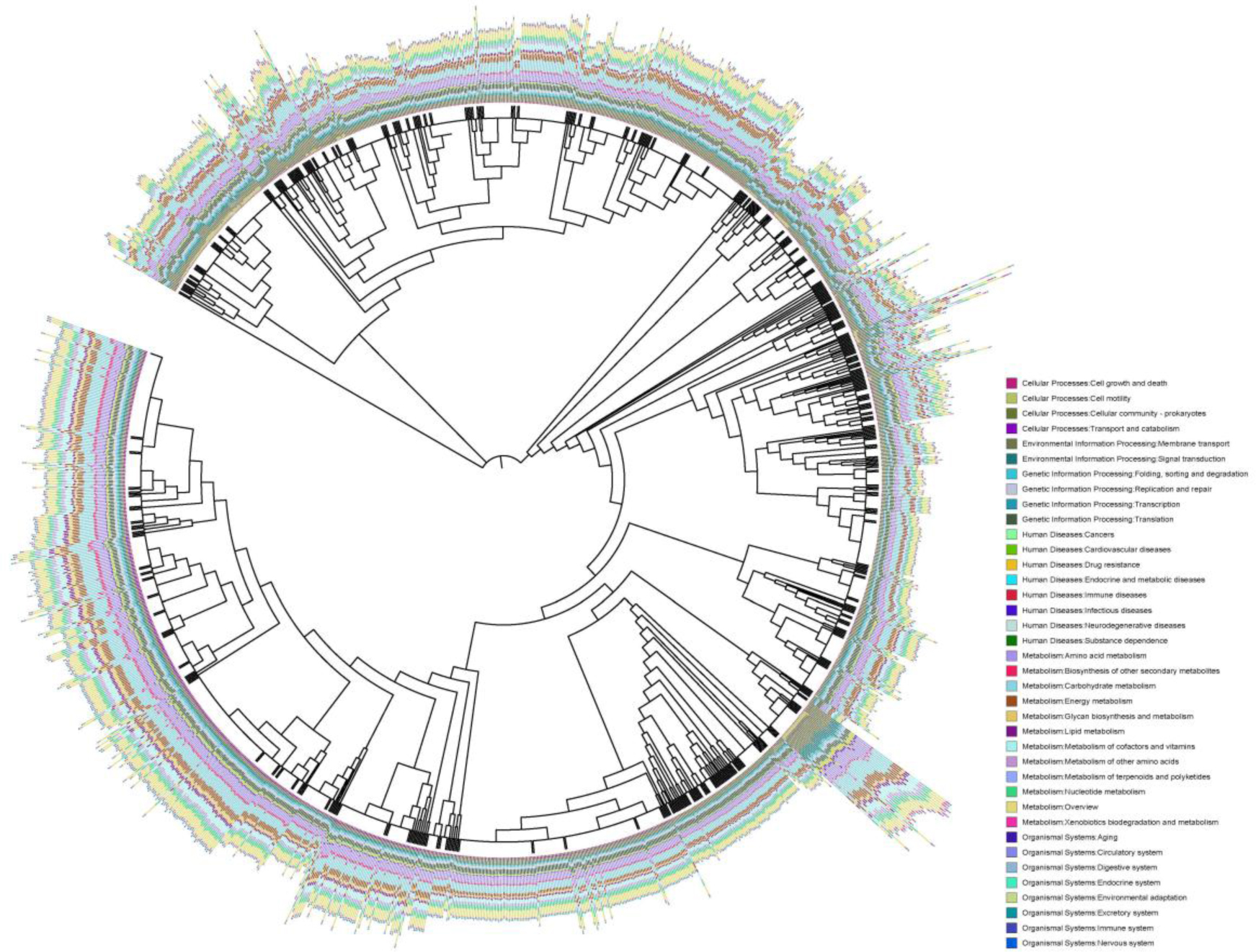

Supplement: Functional annotation of 1,520 genomes in CGR. — The gene functions in the genomes are annotated using KEGG pathways, with level 2 functions shown in the figure. The stack bar on the out-most layer represents the number of genes with given functions in each genome. The phylogenetic tree is plotted according to Fig. 1. [file 41587_2018_8_Fig10_ESM.jpg]

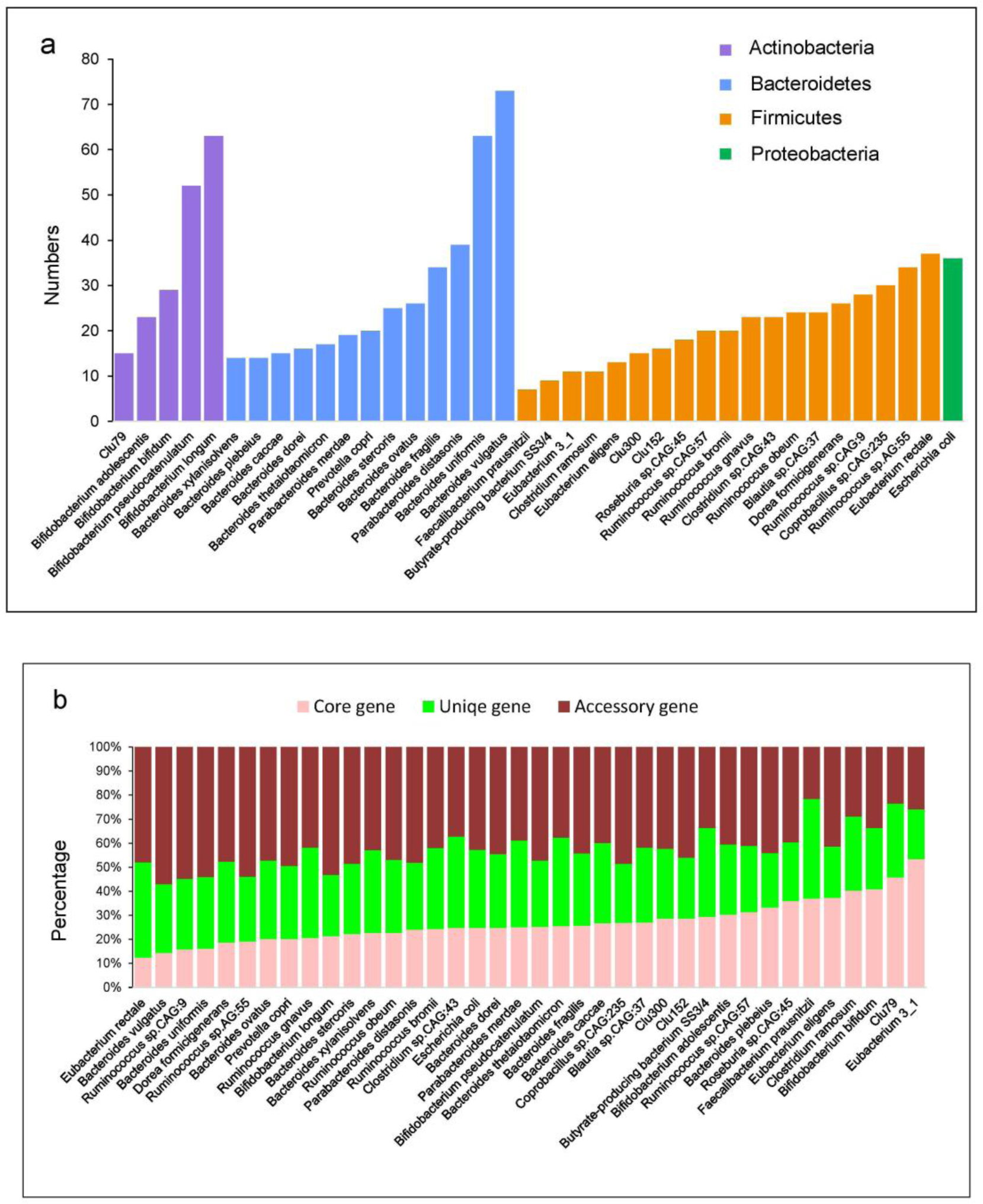

Supplement: Statistics for the pan-genome analysis of the 38 clusters. — (a) Genomes for each cluster used in the pan-genome analysis. (b) Composition of core genes, unique genes, and accessory genes in the genomes of the 38 clusters. The clusters were ordered by the proportion of core genes. [file 41587_2018_8_Fig11_ESM.jpg]

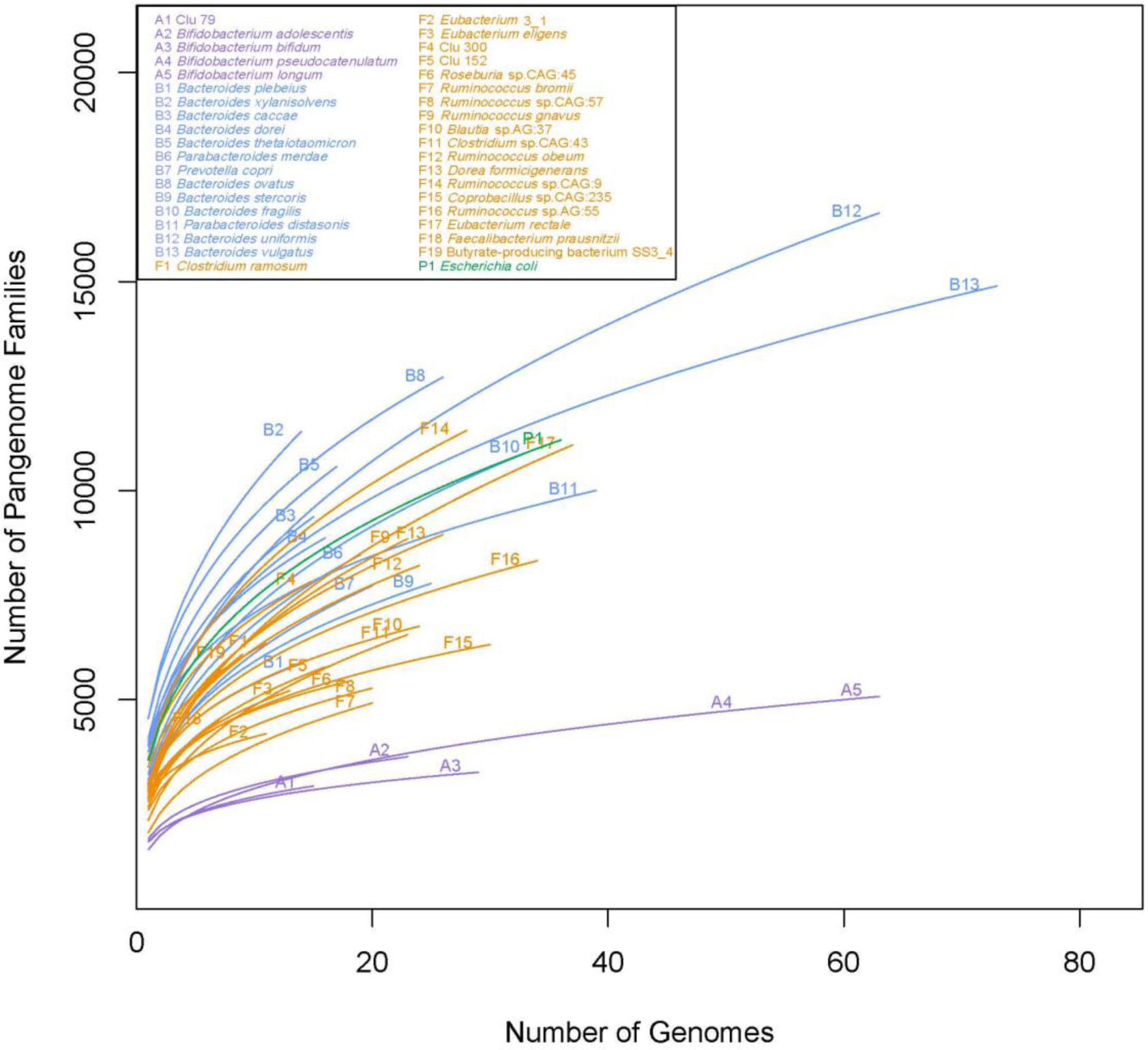

Supplement: Pan-genome fitting curves of the 38 clusters. — The pan-genome fitting curves of 38 representative clusters, from Firmicutes (orange), Bacteroidetes (blue), Actinobacteria (violet), Proteobacteria (green), and Fusobacteria (grey). The pan-genome size is accumulated from all combinations of strains contained in each cluster. [file 41587_2018_8_Fig12_ESM.jpg]

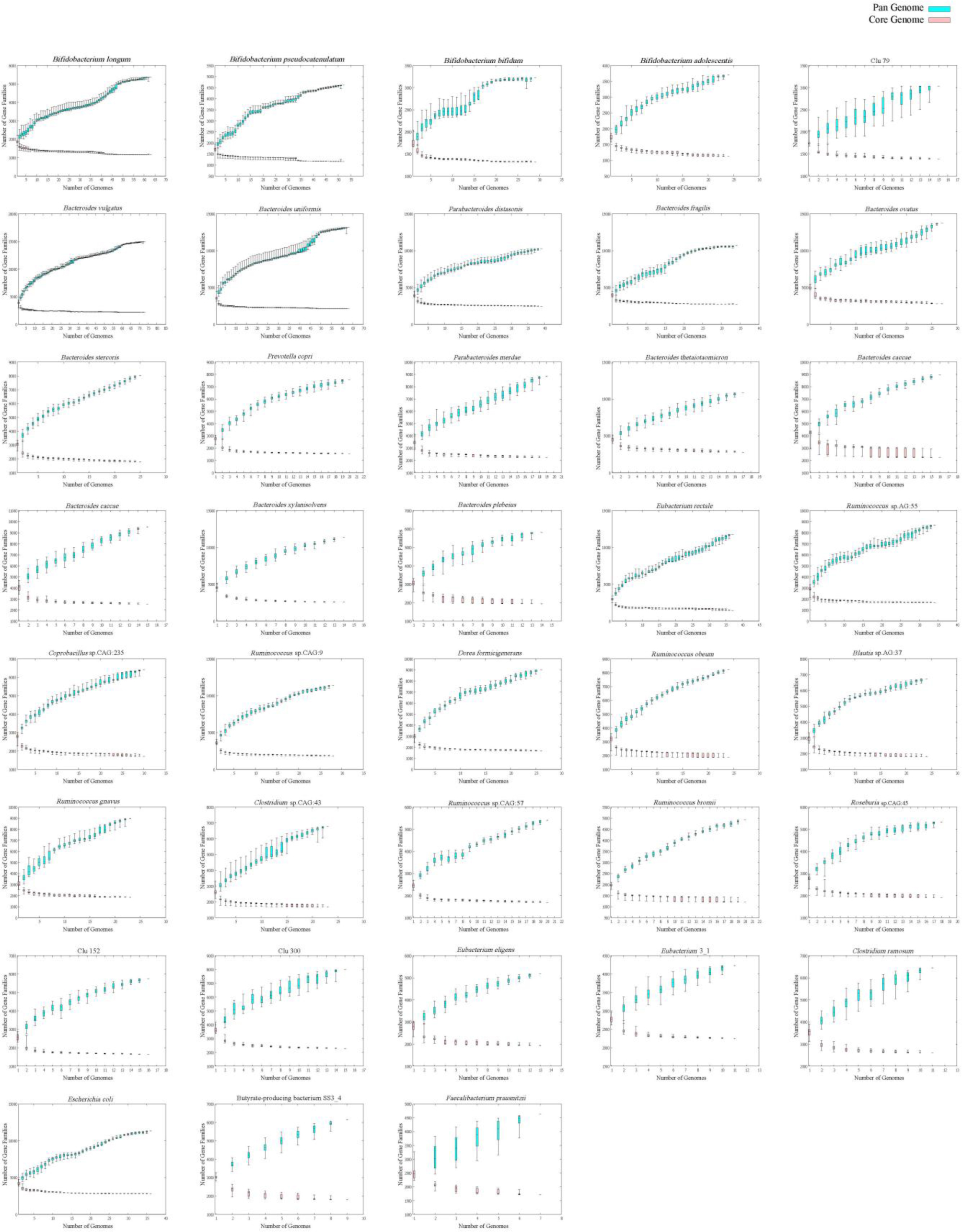

Supplement: Pan- and core-genome analysis of the 38 clusters. — The number of gene families in the pan (cyan) and core (pink) genomes are plotted as a function of the number of genomes of the 38 clusters. Box plots indicate 25 th and 75 th percentiles with medians shown as horizontal lines and whiskers set at 10 th and 90 th percentiles. [file 41587_2018_8_Fig13_ESM.jpg]

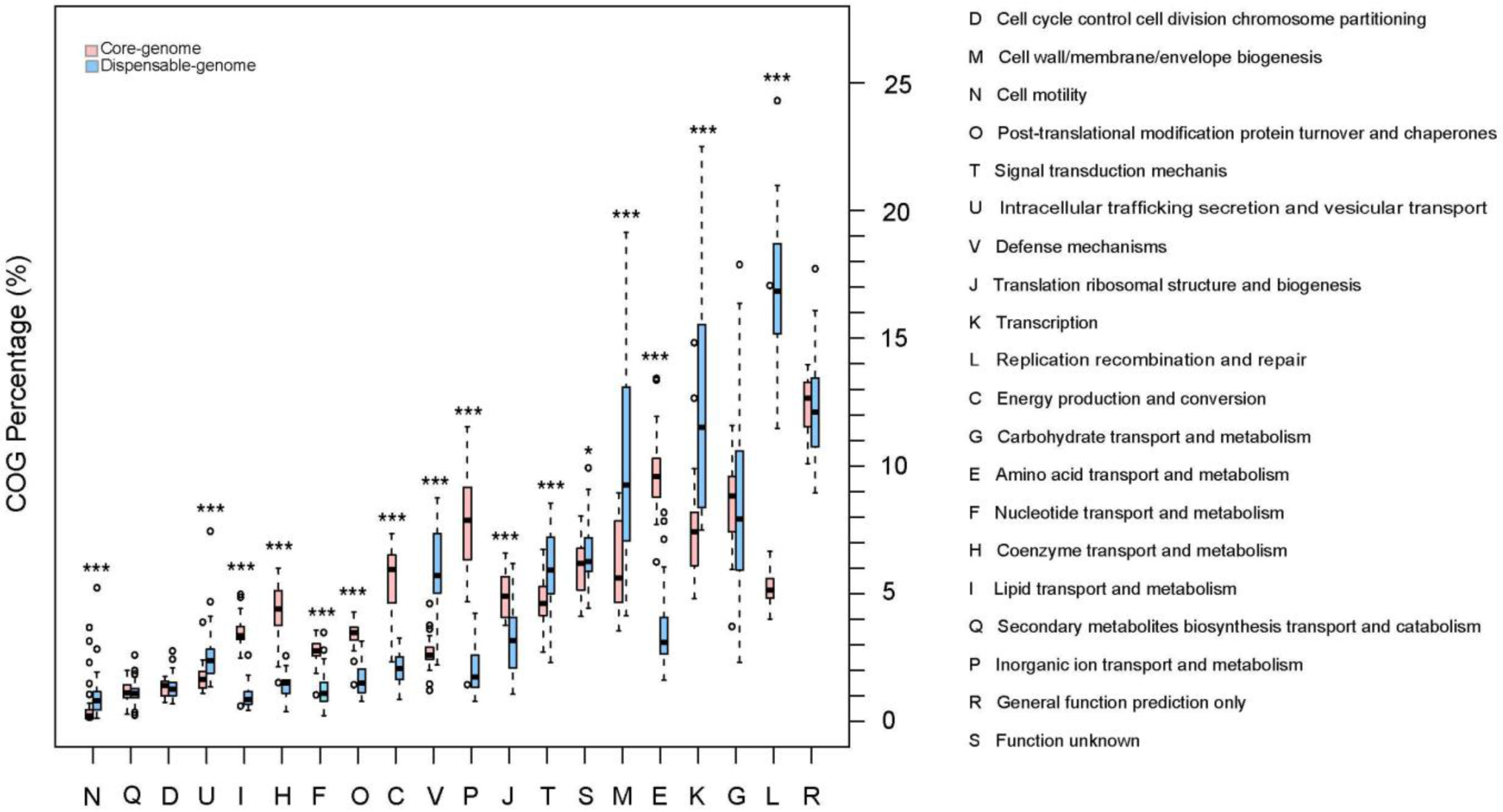

Supplement: COG distribution in the core genome and the dispensable genome. — The percentage of 20 COGs in the core genome (pink) was compared to that in the pan-genomes (cyan) of 38 clusters. The significance of improvement was determined by two-side Wilcoxon rank-sum test (*,P< 0.05; **, P < 0.01; ***, P < 0.001). The exact P value is 0.931 for D, 2.70×10^-9^ for M, 3.11×10^-5^ for N, 7.28×10^-12^ for O, 3.88×10^-4^ for T, 1.22×10^-7^ for U, 7.28×10^-12^ for V, 7.28×10^-12^ for J, 3.64×10^-11^ for K, 7.28×10^-12^ for L, 7.28×10^-12^ for C, 0.261 for G, 7.28×10^-12^ for E, 7.28×10^-12^ for F, 1.46×10^-11^ for H, 2.40×10^-10^ for I, 0.874 for Q, 2.40×10^-10^ for P, 0.365 for R, and 0.031 for S. Each boxplot illustrates the estimated median (centre line), upper and lower quartiles (box limits), 1.5 × interquartile range (whiskers), and outlier (points) of the COG percentage. [file 41587_2018_8_Fig14_ESM.jpg]
