# Supplementary material for: 1,520 reference genomes from cultivated human gut bacteria enable functional microbiome analyses
Source: Nat Biotechnol. 2019 Feb 4;37(2):179–85. doi: 10.1038/s41587-018-0008-8 (PMC6784896; doi:10.1038/s41587-018-0008-8)
Supplement: Supplementary file 11 — Supplementary Figures 1–10 [file 41587_2018_8_MOESM1_ESM.pdf]

nature biotechnology

SUPPLEMENTARY INFORMATION
https://doi.org/10.1038/s41587-018-0008-8

In the format provided by the authors and unedited.

1,520 reference genomes from cultivated human gut bacteria enable functional microbiome analyses

Yuanqiang Zou[1,2,3,13], Wenbin Xue[1,2,13], Guangwen Luo[1,2,4,13], Ziqing Deng[1,2,13], Panpan Qin[1,2,5,13], Ruijin Guo[1,2], Haipeng Sun[1,2], Yan Xia[1,2,5], Suisha Liang[1,2,6], Ying Dai[1,2], Daiwei Wan[1,2], Rongrong Jiang[1,2], Lili Su[1,2], Qiang Feng[1,2], Zhuye Jie[1,2], Tongkun Guo[1,2], Zhongkui Xia[1,2], Chuan Liu[1,2,6], Jinghong Yu[1,2], Yuxiang Lin[1,2], Shanmei Tang[1,2], Guicheng Huo[4], Xun Xu[1,2], Yong Hou[1,2], Xin Liu[1,2,7], Jian Wang[1,8], Huanming Yang[1,8], Karsten Kristiansen[1,2,3,9], Junhua Li[1,2,10]*, Huijue Jia[1,2,11]* and Liang Xiao[1,2,6,9,12]*

[1]BGI-Shenzhen, Shenzhen, China. [2]China National Genebank, BGI-Shenzhen, Shenzhen, China. [3]Laboratory of Genomics and Molecular Biomedicine, Department of Biology, University of Copenhagen, Copenhagen, Denmark. [4]Key Laboratory of Dairy Science, College of Food Sciences, Northeast Agricultural University, Harbin, Heilongjiang, China. [5]BGI Education Center, University of Chinese Academy of Sciences, Shenzhen, China. [6]Shenzhen Engineering Laboratory of Detection and Intervention of Human Intestinal Microbiome, Shenzhen, China. [7]BGI-Qingdao, BGI-Shenzhen, Qingdao, China. [8]James D. Watson Institute of Genome Sciences, Hangzhou, China. [9]Qingdao-Europe Advanced Institute for Life Sciences, Qingdao, China. [10]School of Bioscience and Biotechnology, South China University of Technology, Guangzhou, China. [11]Macau University of Science and Technology, Taipa, Macau, China. [12]Department of Digestive Diseases, Huashan Hospital of Fudan University, Shanghai, China. [13]These authors contributed equally: Yuanqiang Zou, Wenbin Xue, Guangwen Luo, Ziqing Deng, Panpan Qin. *e-mail: lijunhua@genomics.cn; jiahuijue@genomics.cn; xiaoliang@genomics.cn

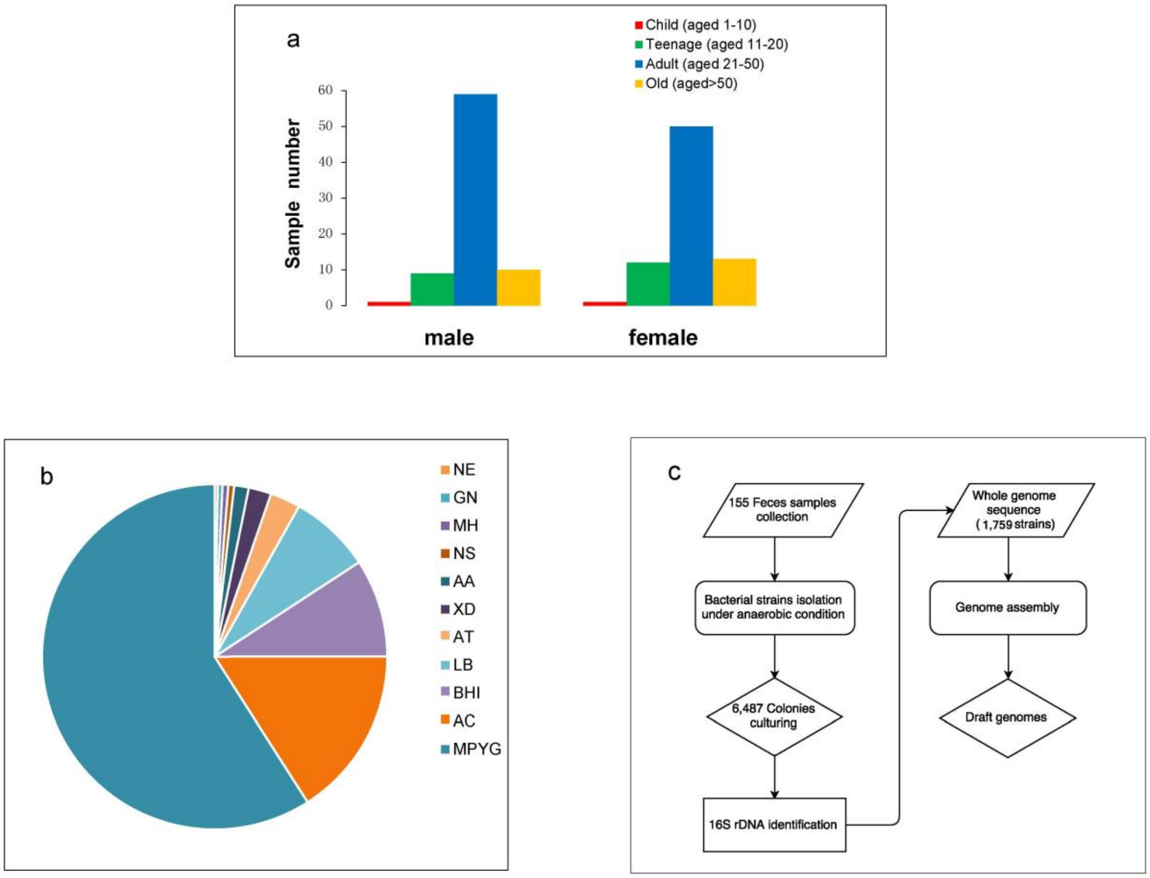

Supplementary Figure 1

Cultivation and genome sequencing of the gut microbiota

(a) The 155 feces samples from healthy volunteers grouped by ages and sex.

(b) The number of isolates achieved by 11 different culture media under anaerobic condition.

(c) The workflow of the cultivation and sequencing of isolated gut bacteria.

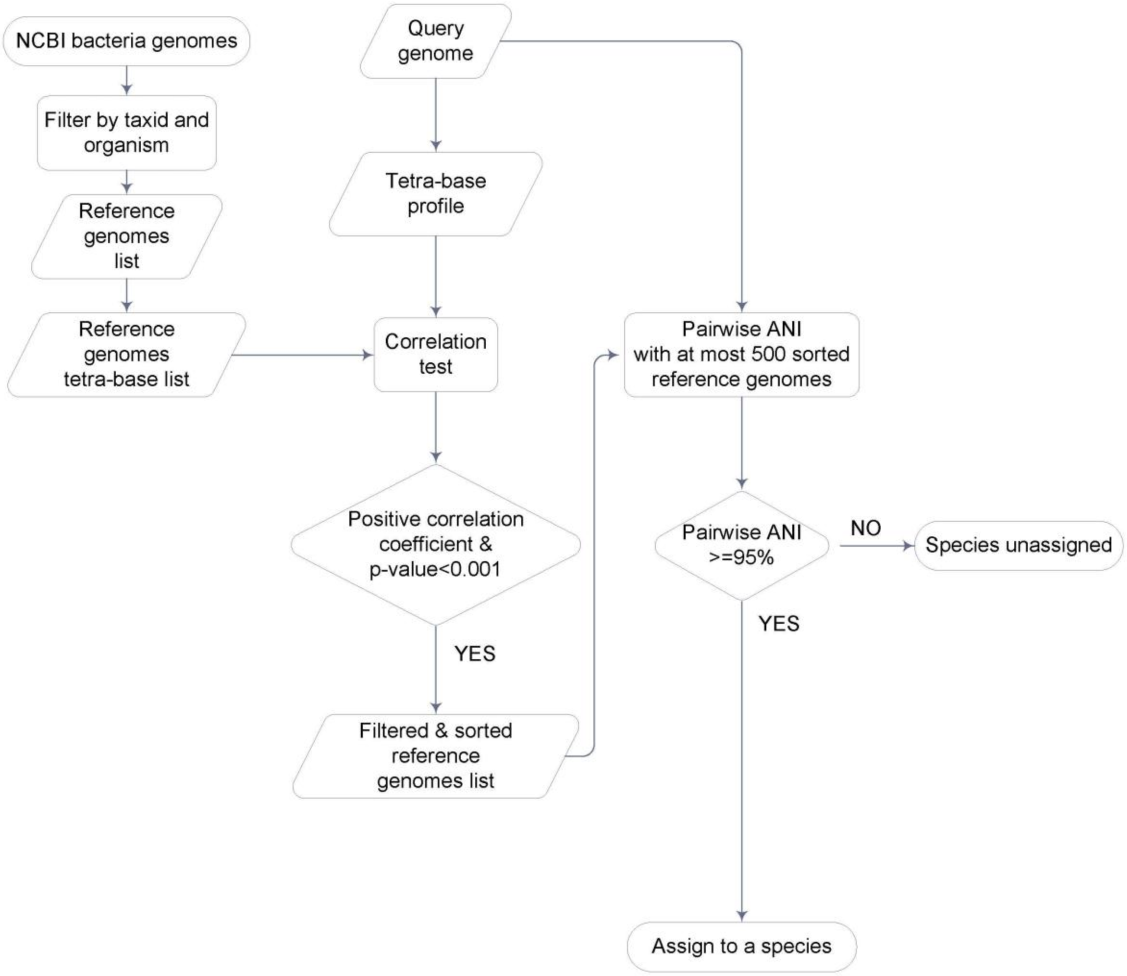

Supplementary Figure 2

Workflow for species annotation of sequenced genomes

Species assignment was carried out using an average-nucleotide identity (ANI)-based pipeline. Genomes not assigned by ANI were subjected to genus annotation by POCP

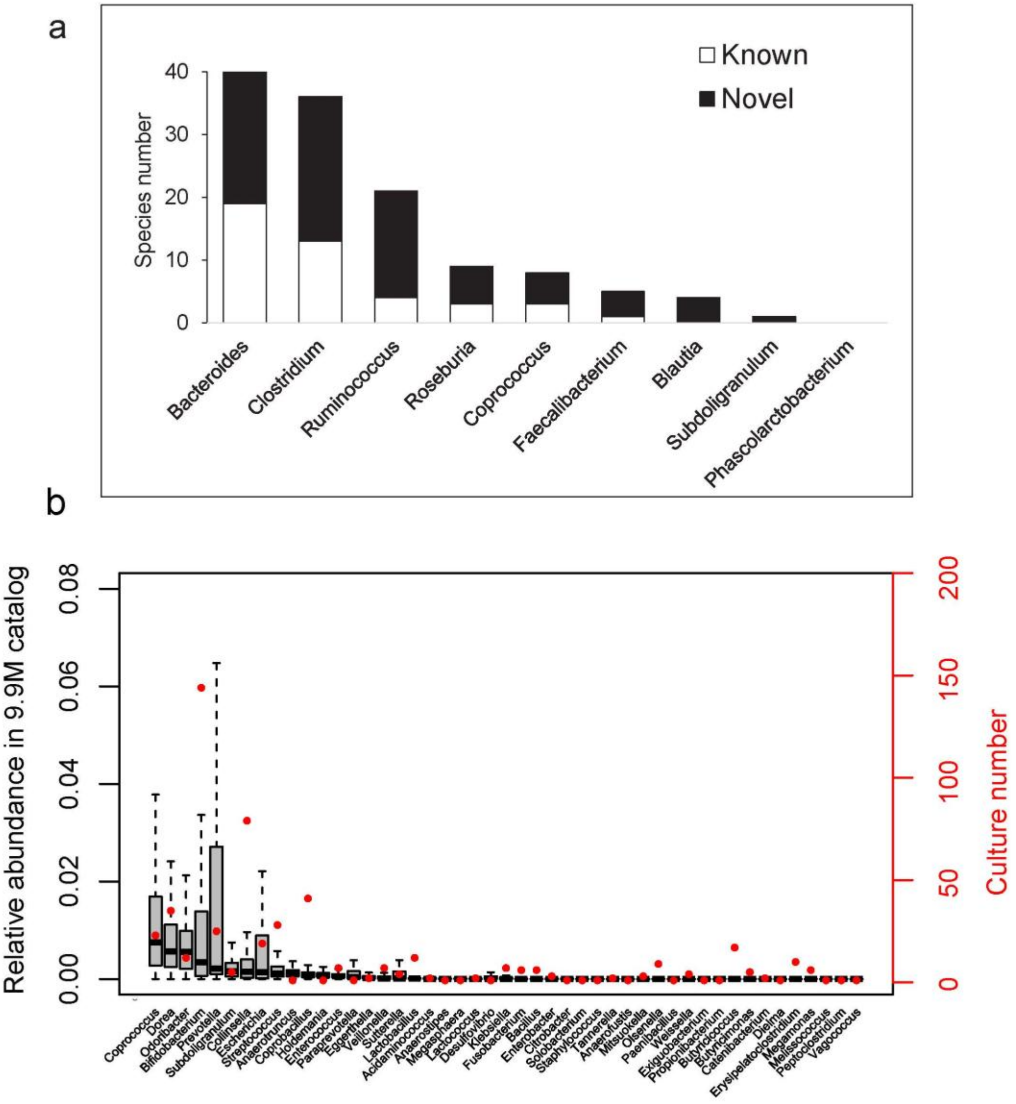

Supplementary Figure 3

Diversity and novelty of gut bacterial genomes archived in CGR

(a) The number of bacterial species archived in CGR belonging to 9 core genera of the human gut microbiota in Chinese. The archived bacterial species were compared to the previously reported 1,000 cultured bacterial species in the human gastrointestinal tract, with the known species shown in white and novel species shown in black.

(b) Low abundance (<1%) gut bacterial genera identified in CGR. Grey box indicates the relative abundance of each genus from 1267 samples, according to the previous IGC study. Red dot indicates the number of species in each genus archived in this study. Each boxplot illustrates the estimated median (centre line), upper and lower quartiles (box limits), 1.5 × interquartile range (whiskers)

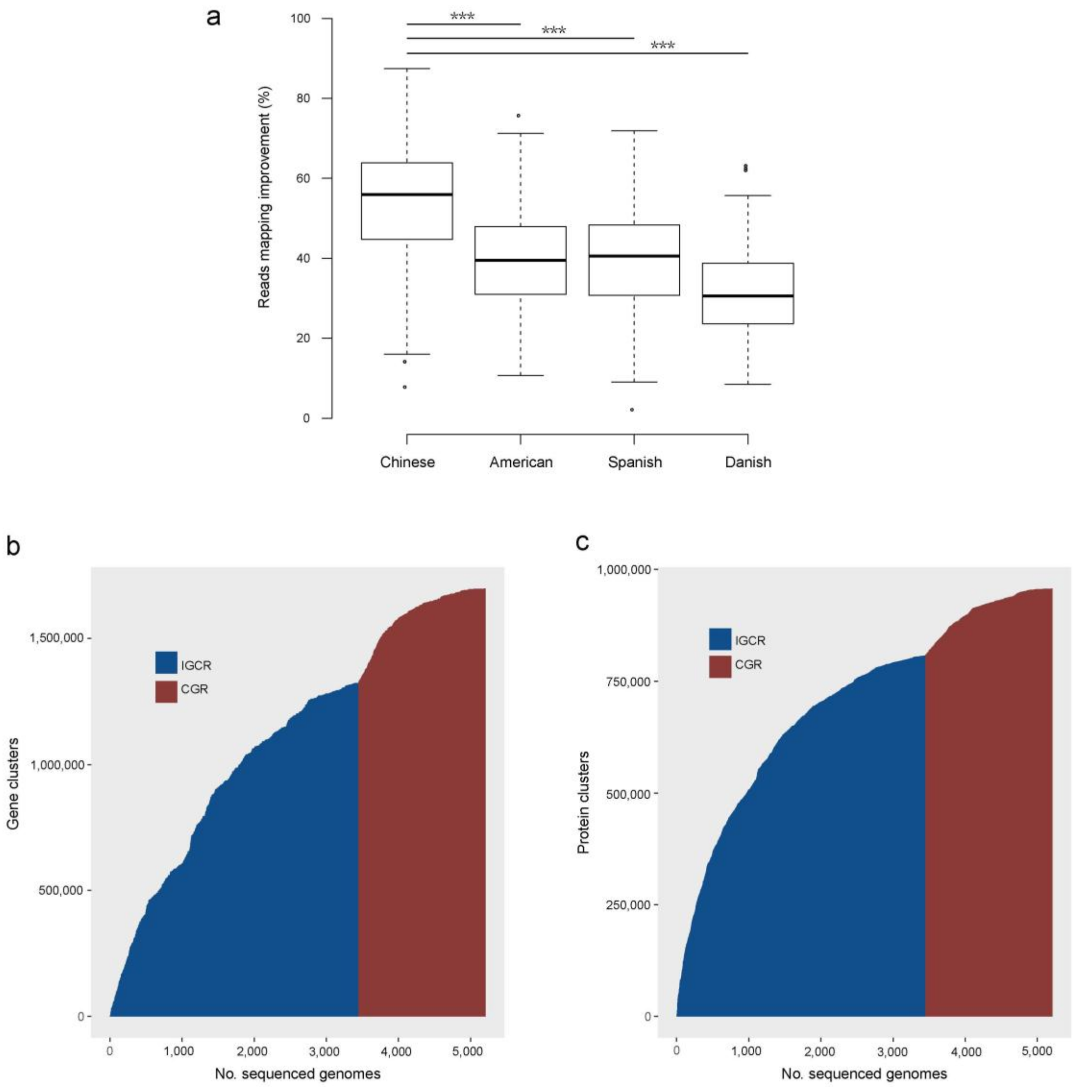

Supplementary Figure 4

The improvement in metagenomic analysis by CGR

(a) The improvement of reads mapping ratio in metagenomic analysis by CGR (relevant to Figure 2a). The percentage of improvement is calculated by the following formula: (CGR-ICG)/(100-ICG). The percentage of improvement for Chinese (n=368) is significantly higher than American (n=139, $P=8\times10^{-20}$), Spanish (n=320, $P=9\times10^{-33}$), and Danish (n=109, $P=2\times10^{-31}$) individuals. The significance of improvement was determined by unpaired Wilcoxon rank-sum test (two.sided). ICG represents the reads mapping ratio calculated from 3,449 reference genomes (ICGR in Figure 2a), CGR represents the reads mapping ratio calculated from the addition of 1,520 reference genomes (ICGR+CGR in Figure 2a). Each boxplot illustrates the estimated median (centre line), upper and lower quartiles (box limits), 1.5 × interquartile range (whiskers), and outlier (points) of the reads mapping ratio.

(b)(c) Gene and protein sequence diversity increased by CGR. Increase in number of new gene families (b) and protein families (c) across added genomes from ICGR (blue) and CGR (red).

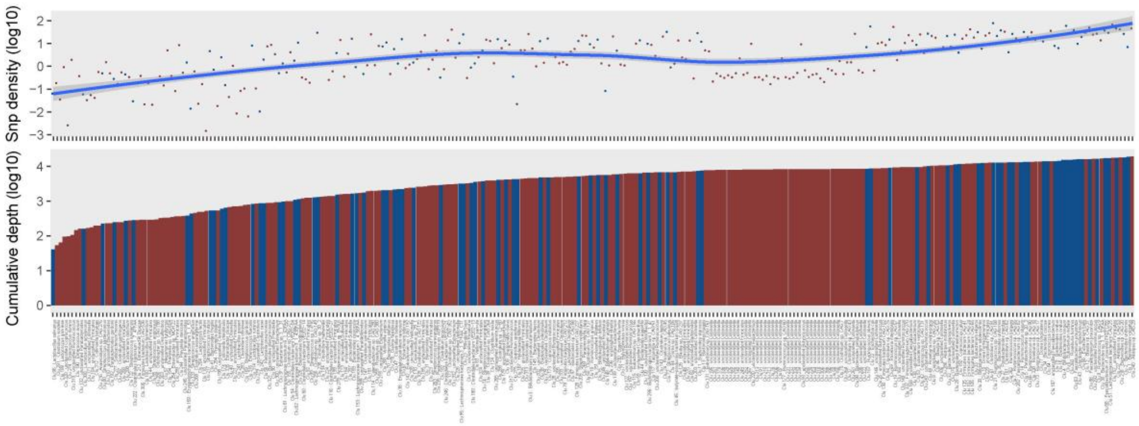

Supplementary Figure 5

SNP density in the 282 reference genomes with a cumulative coverage of at least 10× in the 250 samples from the TwinsUK registry.

The reference genomes are ordered according to the cumulative coverage, with new reference genomes generated by this study highlighted in red.

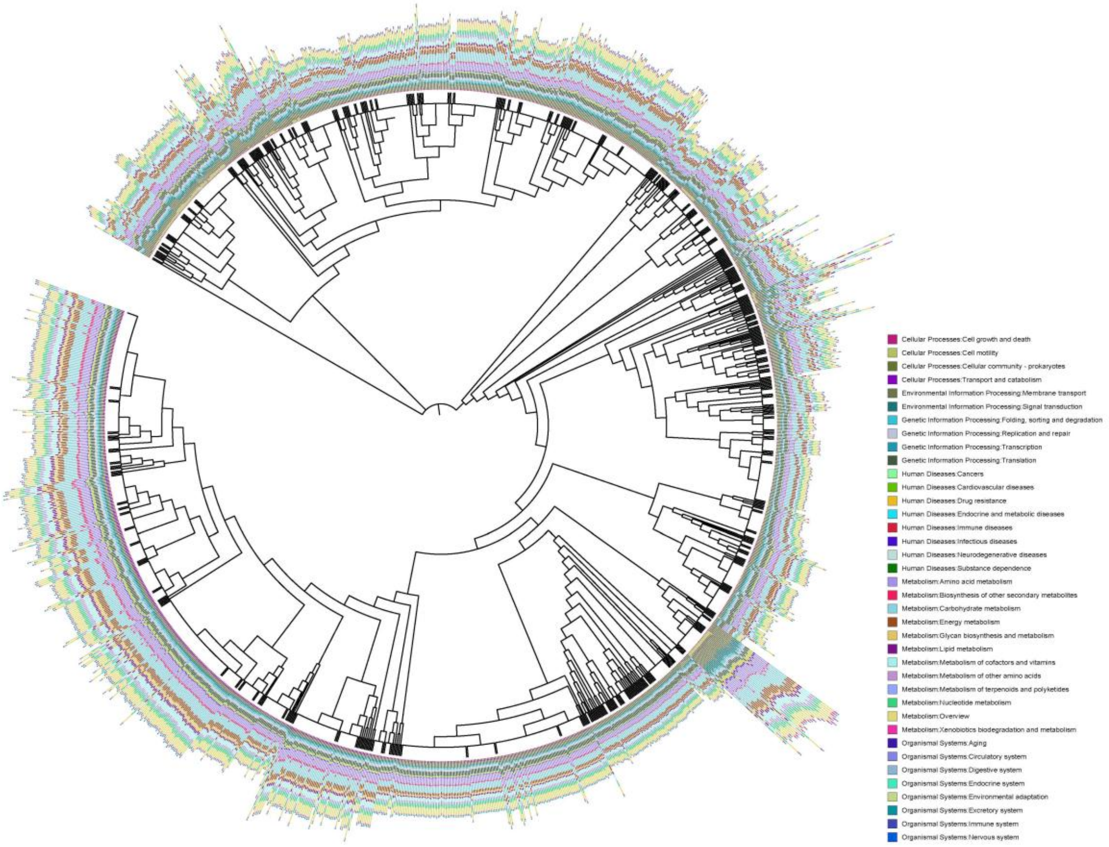

Supplementary Figure 6

Functional annotation of 1,520 genomes in CGR

The gene functions in the genomes are annotated using KEGG pathways, with level 2 functions shown in the figure. The stack bar on the out-most layer represents the number of genes with given functions in each genome. The phylogenetic tree is plotted according to Figure 1.

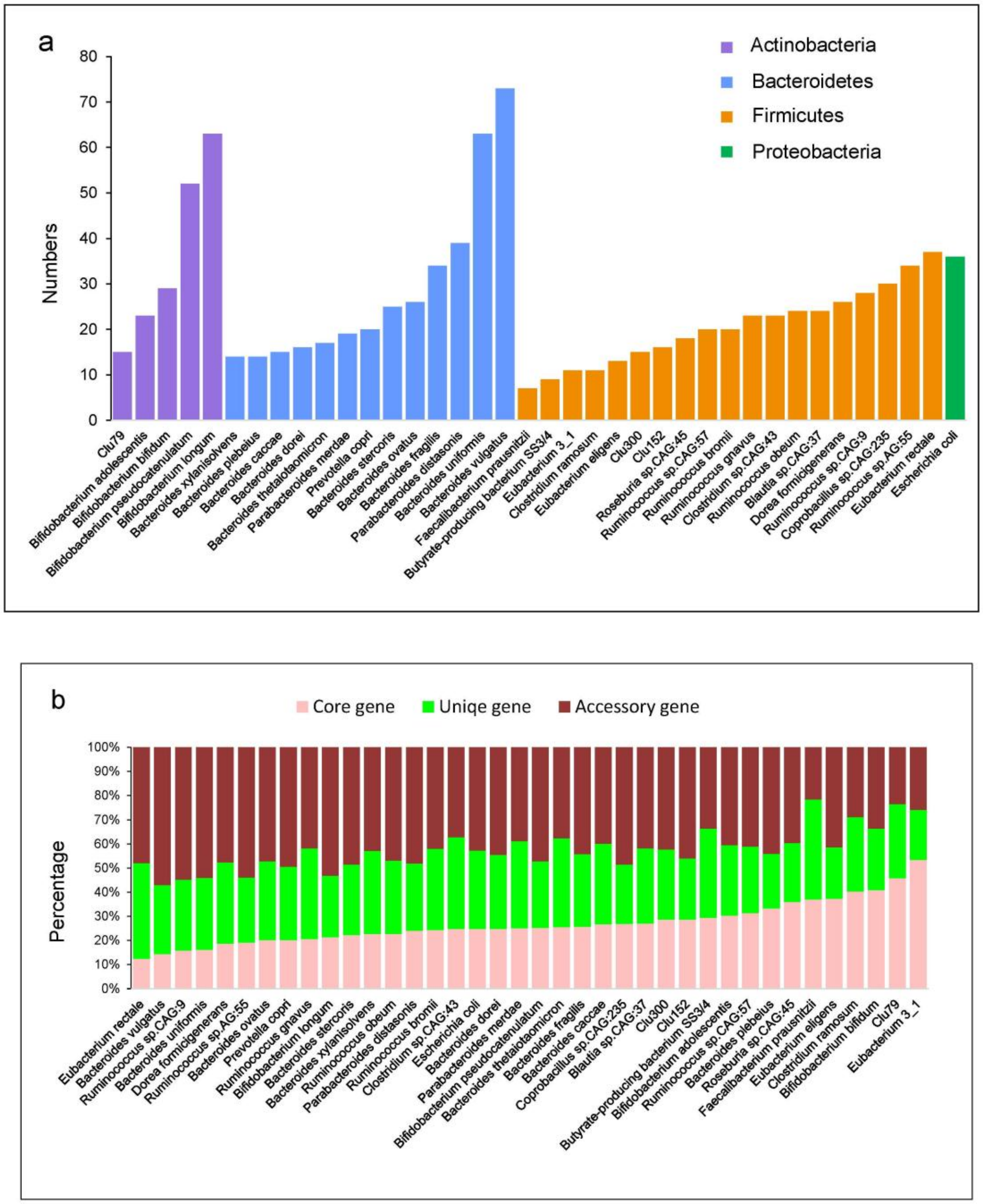

Supplementary Figure 7

Statistics for the pan-genome analysis of the 38 clusters.

(a) Genomes for each cluster used in the pan-genome analysis.

(b) Composition of core genes, unique genes, and accessory genes in the genomes of the 38 clusters. The clusters were ordered by the proportion of core genes.

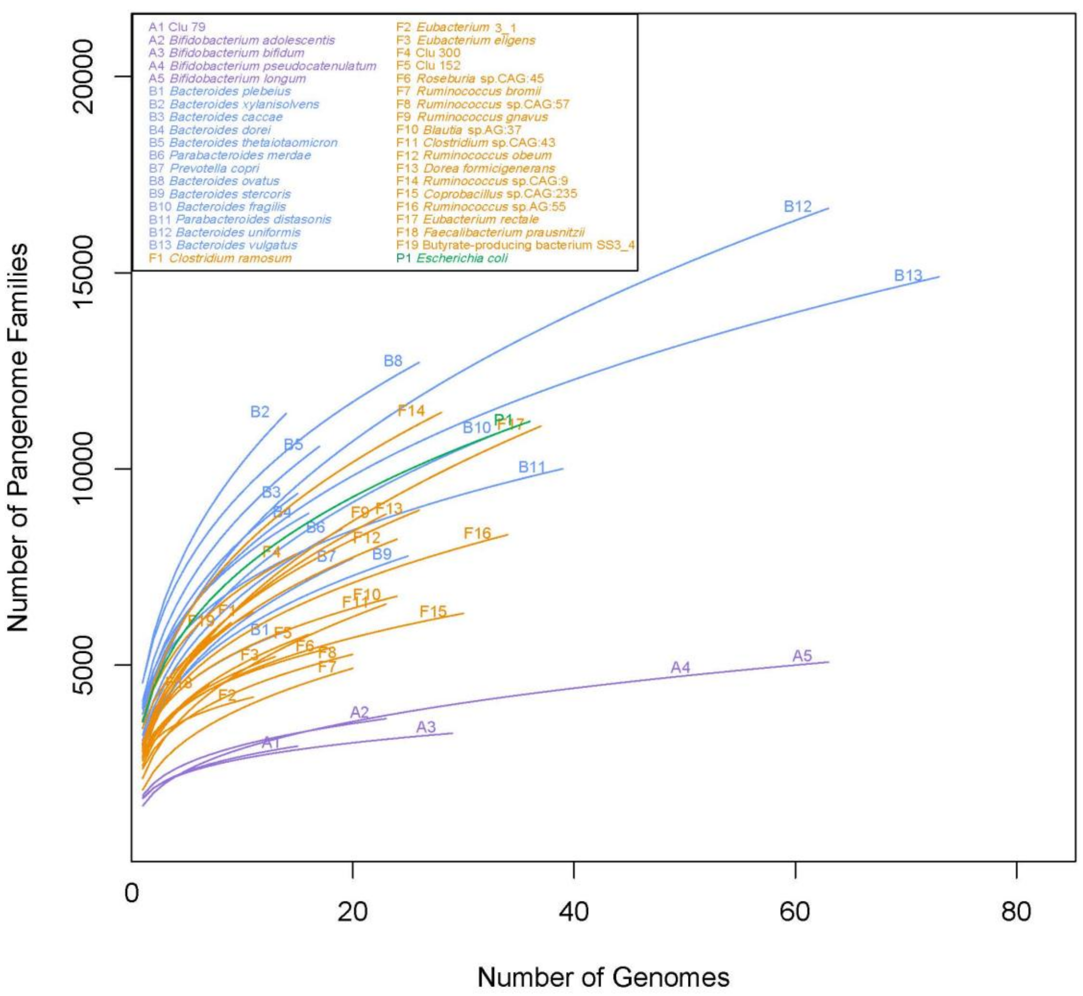

Supplementary Figure 8

Pan-genome fitting curves of the 38 clusters

The pan-genome fitting curves of 38 representative clusters, from Firmicutes (orange), Bacteroidetes (blue), Actinobacteria (violet), Proteobacteria (green), and Fusobacteria (grey). The pan-genome size is accumulated from all combinations of strains contained in each cluster.

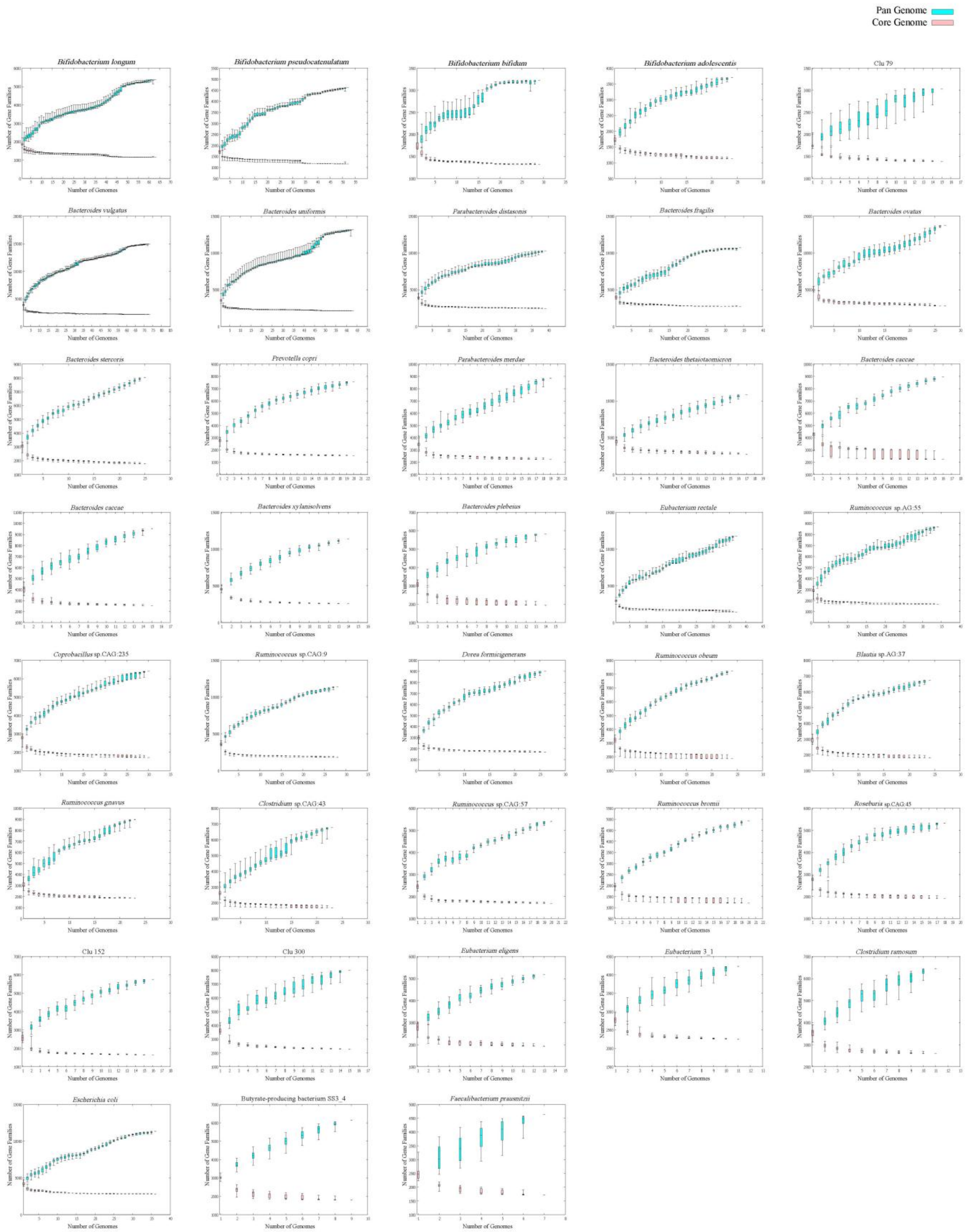
Pan Genome
Core Genome
Bifidobacterium longum
Bifidobacterium pseudocatenulatum
Bifidobacterium bifidum
Bifidobacterium adolescentis
Clu 79
Bacteroides vulgatus
Bacteroides uniformis
Parabacteroides distasonis
Bacteroides fragilis
Bacteroides ovatus
Bacteroides stercoris
Prevotella copri
Parabacteroides merdae
Bacteroides thetaiotaomicron
Bacteroides caccae
Bacteroides caccae
Bacteroides xylanisolvens
Bacteroides plebeius
Eubacterium rectale
Ruminococcus sp.AG:55
Coprobacillus sp.CAG:235
Ruminococcus sp.CAG:9
Dorea formicigenerans
Ruminococcus obeum
Blautia sp.AG:37
Ruminococcus gnavus
Clostridium sp.CAG:43
Ruminococcus sp.CAG:57
Ruminococcus bromii
Roseburia sp.CAG:45
Clu 152
Clu 300
Eubacterium eligens
Eubacterium 3_1
Clostridium ramosum
Escherichia coli
Butyrate-producing bacterium SS3_4
Faecalibacterium prausnitzii
Number of Gene Families
Number of Genomes

Supplementary Figure 9

Pan- and core-genome analysis of the 38 clusters

The number of gene families in the pan (cyan) and core (pink) genomes are plotted as a function of the number of genomes of the 38 clusters. Box plots indicate 25 th and 75 th percentiles with medians shown as horizontal lines and whiskers set at 10 th and 90 th percentiles

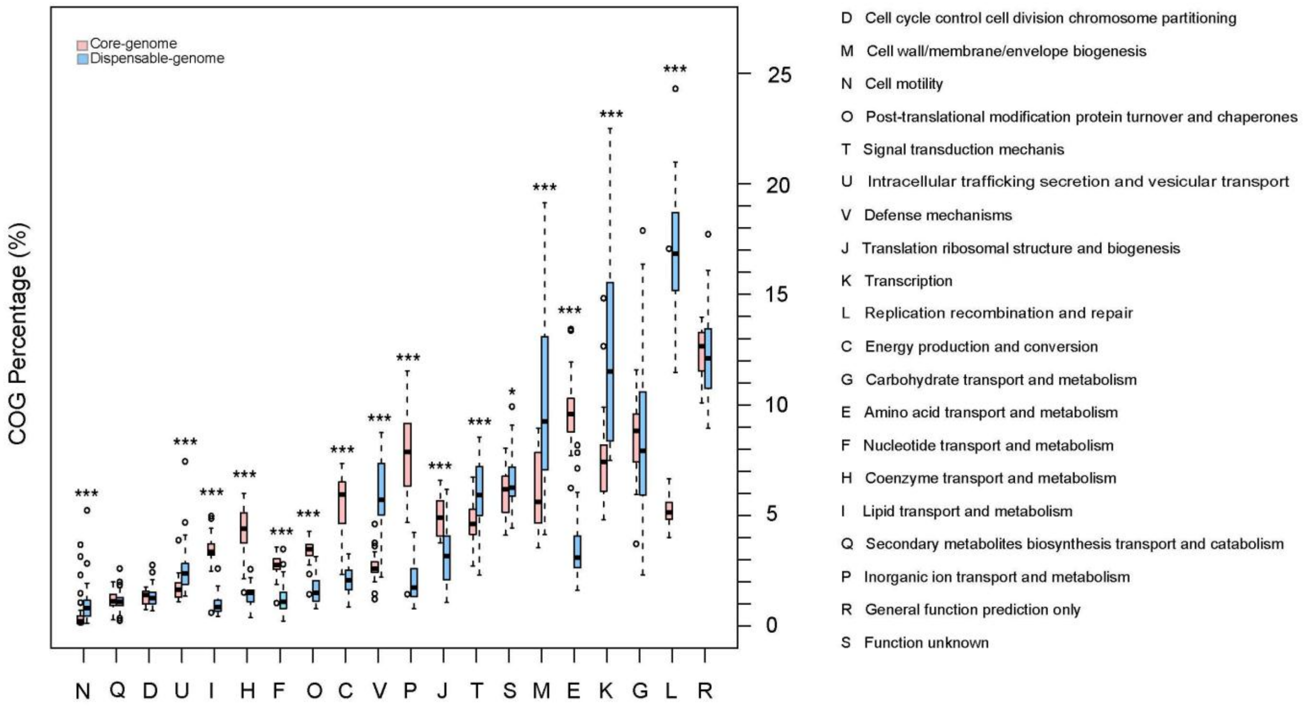

Supplementary Figure 10

COG distribution in the core genome and the dispensable genome

The percentage of 20 COGs in the core genome (pink) was compared to that in the pan-genomes (cyan) of 38 clusters. The significance of improvement was determined by two-side Wilcoxon rank-sum test (*,P< 0.05; **, P < 0.01; ***, P < 0.001). The exact P value is 0.931 for D, 2.70×10^{-9} for M, 3.11×10^{-5} for N, 7.28×10^{-12} for O, 3.88×10^{-4} for T, 1.22×10^{-7} for U, 7.28×10^{-12} for V, 7.28×10^{-12} for J, 3.64×10^{-11} for K, 7.28×10^{-12} for L, 7.28×10^{-12} for C, 0.261 for G, 7.28×10^{-12} for E, 7.28×10^{-12} for F, 1.46×10^{-11} for H, 2.40×10^{-10} for I, 0.874 for Q, 2.40×10^{-10} for P, 0.365 for R, and 0.031 for S. Each boxplot illustrates the estimated median (centre line), upper and lower quartiles (box limits), 1.5 × interquartile range (whiskers), and outlier (points) of the COG percentage.
